# Supplementary material for: Repurposing the orphan drug nitisinone to control the transmission of African trypanosomiasis
Source: PLoS Biol. 2021 Jan 26;19(1):e3000796. doi: 10.1371/journal.pbio.3000796 (PMC7837477; doi:10.1371/journal.pbio.3000796)
Supplement: S1 Methods — Mathematical modelling for T. brucei gambiense transmission. (DOCX) [file pbio.3000796.s001.docx]

**Supplementary Methods**

**Mathematical modelling for *Trypanosoma brucei* rhodesiense transmission**

The model we have selected to determine feasibility of HAT control with the novel endectocide is analogous to the Ross-Macdonald model, which has played a central role in the development of research on mosquito-borne pathogen transmission and the development of strategies for mosquito-borne disease prevention [1]. This simplistic and transparent modelling is more appropriate for the current, early, proof-of-principle stage. Later in its development, more biologically realistic models will become more appropriate tools for assessing NTBC deployment strategy in specific settings [2,3].

A discrete time (1-day time step) compartmental model was constructed to describe the key processes underlying the transmission of *T. b. rhodesiense*. This parasite has a broad reservoir of host animals so these were categorised according to whether they comprised livestock or wildlife. The transmission between three host types in total (humans, livestock and wildlife abbreviated below to host group i) and the tsetse fly vector was tracked according to the following set of equations:

$$S_{t+1}^{i}={1-I}_{t}^{i}$$

$$I_{t+1}^{i}=I_{t}^{i}+\frac{V}{N_{i}}p_{i}b_{i}Z_{t}S_{t}^{i}-\left( g_{i}+m_{i} \right)I_{t}^{i}$$

$$X_{t+1}=1-(Y_{t}+Z_{t})$$

$$Y_{t+1}=Y_{t}+\sum_{i} I_{t}^{i}{X_{t}p}_{i}b_{vi}-\left( d+m_{1}+m_{2}+m_{3} \right)Y_{t}$$

$$Z_{t+1}=Z_{t}+dY_{t}-{(m}_{1}+m_{2}+m_{3})Z_{t}$$

Here, susceptible hosts (*S*) become infected (*I*) at a rate dependent on the ratio of vectors (*V*) to hosts in that group (*N*); the proportion of the bites tsetse flies make on that host group (*p*, e.g. pH is the proportion of bites on ‘H’umans, also called the human blood index [4]); the bite rate of the vector multiplied by the probability that an infectious bite will successfully infect the host (*b*); the proportion of vectors that are infectious (*Z*) and the proportion of hosts that are susceptible to infection. Infected hosts then recover (at rate *g*) or die (at rate *m*). Similarly, susceptible vectors (*X*) become infected (*Y*) dependent on the proportion of bites tsetse flies make on that host group; the bite rate of the vector multiplied by the probability that an infectious bite will successfully infect the vector (*bv*); and the proportion of vectors that are susceptible and hosts that are infectious. For the force of infection in the vector population, these rates must be summed across the three groups of hosts. To account for the extrinsic incubation period, the infected vectors become infectious at rate *d*. Vector death can happen any of three ways: natural mortality rate *m1*; through biting a human host using NTBC, *m2*­; or through biting a livestock host treated with NTBC, *m3*. NTBC efficacy wanes with time (we have conservatively assumed a 7-day half-life), and this is accounted for using the following equation:

$$m_{2}=\psi.\exp\left( -T.\lambda\right)p_{H}\times coverage level\times bite rate$$

Whereby *m2­* is the waning, additional mortality incurred by tsetse flies biting humans treated with NTBC; *ψ* is the maximum tsetse lethality (here assumed 100%) when a blood-meal is taken from a very-recently treated human; *T* is the number of days since treatment and *λ* determines the efficacy half-life. An analogous equation is used to simulate declining NTBC efficacy when used on livestock (*m3*). Vector biting behaviour is examined by apportioning the split of bites on humans, livestock and wildlife randomly across the full possible range and, through simulation, estimating the impact NTBC application has on the effective reproduction number (*Re*). As the killing efficacy decreases between endectocidal applications, *Re* returns to its pre-control level (i.e., R0). Therefore, the *Re* as plotted in Fig 5 (for *T. b. rhodesiense*) and in Fig S15 (for *T. b. gambiense*) is the *Re* averaged over the treatment cycle. To show how these summary plots relate to temporal dynamics, we have plotted example dynamics for when *T. b. rhodesiense* is controlled using monthly dosing of both humans and livestock with NTBC. The many lines shown in Fig S14 are the simulated results for a random assortment of bloodmeals (split between humans, livestock and wildlife).

Conservatively, we assumed that residual NTBC effects from previous application rounds were negligible. We also assumed a maximum coverage rate of 80% for both livestock and humans. Parameterisation is detailed in Table A and yields a basic reproduction ratio, *R0*, of 1.1, as reported previously [5].

Table A. Model parameters, definitions, units, values and sourcing literature.

| Parameters | Definition (units) | | Values | Sources |
| --- | --- | --- | --- | --- |
| *p* | Proportion of bites on the host group | All combinations simulated | |  |
| *b* | Bite rate x probability of parasite transmission to host (day^-1^): | ¼ x *see below | | [5,6] |
| *g* | Recovery rate (day^-1^): humans (*T.b.g* and *T.b.r*) | 1.3e-3 & 1.7e-3 | | [7,8] |
|  | livestock | 8.7e-3 | | [9] |
|  | wildlife | 8.7e-3 | | assumed |
| *m* | Mortality rate (day^-1^): humans | 5.5e-5 | | assumed |
|  | livestock | 1.4e-3 | | [5] |
|  | wildlife | 9.1e-5 | | [5] |
| *b­_v_* | Bite rate x probability of parasite transmission to vector (day^-1^): | ¼ x *see below | | [6,10] |
| *d* | Extrinsic incubation period of parasite (day^-1^) | 2.9e-2 | | [6] |
| *ψ* | Maximum tsetse lethality of fresh NTBC treatment | 1.0 | |  |
| *λ* | Efficacy half-life: oral dose in humans | 0.099  (7-day of efficacy) | | This paper |
|  | Oral dose in livestock | 0.099  (7-day of efficacy) | | This paper |

* transmission probabilities were set to ensure R0 = 1.1.

**Mathematical modelling for *T. brucei gambiense* transmission**

Following Funk et al. (2013) [5], the basic reproduction number can be calculated as follows:

$$R_{0}=\sqrt{\sum_{i} R_{i}}$$

Where,

$$R_{i}=\frac{(V/i)b_{i}b_{Vi}p_{i}d}{m_{V}\left( g_{i}+m_{i} \right)\left( m_{V}+d \right)}$$

Assuming *R0* = 1.1, the above equation can be rearranged to give the transmission coefficients:

$$b_{i}b_{Vi}=\frac{1.21\left( m_{V}\left( g_{i}+m_{i} \right)\left( m_{V}+d \right) \right)}{(V/i)p_{i}d}$$

Here, *mv* is the total vector mortality rate according to the different treatment regimens (i.e. natural mortality summed with any additional mortality from endectocide exposure from the associated host species). Measuring the transmission coefficients in reality is often unfeasible, so, following the convention of previous HAT modelling papers [2], we assume *bi* = *bvi*. For *T. brucei gambiense*, we also follow precedence by assuming that transmission coefficients for non-human hosts are zero [2]. However, for *T. brucei rhodesiense*, we assume that the combined contribution of livestock and wildlife equal the contribution of humans to transmission [11].

**Supplementary references**

1. Smith DL, Battle KE, Hay SI, Barker CM, Scott TW, McKenzie FE. Ross, Macdonald, and a theory for the dynamics and control of mosquito-transmitted pathogens. PLoS Pathog. 2012. doi:10.1371/journal.ppat.1002588

2. Rock KS, Ndeffo-Mbah ML, Castaño S, Palmer C, Pandey A, Atkins KE, et al. Assessing strategies against gambiense sleeping sickness through mathematical modeling. Clin Infect Dis 2018: 66;4: S286-S292. doi:10.1093/cid/ciy018

3. Stone CM, Chitnis N. Implications of heterogeneous biting exposure and animal hosts on *Trypanosomiasis brucei gambiense* transmission and control. PLoS Comput Biol. 2015;11. doi:10.1371/journal.pcbi.1004514

4. Garrett-Jones C. The human blood index of malaria vectors in relation to epidemiological assessment. Bull World Health Organ. 1964;30: 241–261.

5. Funk S, Nishiura H, Heesterbeek H, Edmunds WJ, Checchi F. Identifying transmission cycles at the human-animal interface: The role of animal reservoirs in maintaining gambiense human African trypanosomiasis. PLoS Comput Biol. 2013;9. doi:10.1371/journal.pcbi.1002855

6. Davis S, Aksoy S, Galvani A. A global sensitivity analysis for African sleeping sickness. Parasitology. 2011;138: 516–526. doi:10.1017/S0031182010001496

7. Checchi F, Funk S, Chandramohan D, Haydon DT, Chappuis F. Updated estimate of the duration of the meningo-encephalitic stage in gambiense human African trypanosomiasis. BMC Res Notes. 2015;8: 292. doi:10.1186/s13104-015-1244-3

8. Odiit, M, F Kansiime JCE. Duration of symptoms and case fatality of sleeping sickness caused by *Trypanosoma brucei* *rhodesiense* in Tororo, Uganda. East Afr Med J. 1997;74: 792–5.

9. Penchenier L, Alhadji D, Bahébégué S, Simo G, Laveissière C, Cuny G. Spontaneous cure of domestic pigs experimentally infected by *Trypanosoma brucei gambiense*: Implications for the control of sleeping sickness. Vet Parasitol. 2005;133: 7–11. doi:10.1016/j.vetpar.2005.04.034

10. Haines LR, Lehane SM, Pearson TW, Lehane MJ. Tsetse EP protein protects the fly midgut from trypanosome establishment. PLoS Pathog. 2010;6: e1000793. doi:10.1371/journal.ppat.1000793

11. Welburn SC, Coleman PG, Maudlin I, Fèvre EM, Odiit M, Eisler MC. Crisis, what crisis? Control of Rhodesian sleeping sickness. Trends in Parasitology; 2006. pp. 123–128. doi:10.1016/j.pt.2006.01.011
