# Supplementary material for: Repurposing the orphan drug nitisinone to control the transmission of African trypanosomiasis
Source: PLoS Biol. 2021 Jan 26;19(1):e3000796. doi: 10.1371/journal.pbio.3000796 (PMC7837477; doi:10.1371/journal.pbio.3000796)
Supplement: S1 Table — Tsetse survival after (A) bloodmeal supplementation with HPLA concentrations or (B) injecting HPLA concentrations into the haemocoel. One independent replicate was performed per experiment; (A) n = 25 flies fed/concentration, (B) n = 10 flies injected/dose. (DOCX) [file pbio.3000796.s002.docx]

**S1 Table.** Tsetse survival after (A) bloodmeal supplementation with HPLA concentrations or (B) injecting HPLA concentrations into the haemocoel. One independent replicate was performed per experiment; (A) n=25 flies fed/concentration, (B) n=10 flies injected/dose.

| **Experiment** | **HPLA Concentrations**  **(mg/ml)** | **Observed fly**  **mortality** | **Total number**  **flies tested** |
| --- | --- | --- | --- |
| (A) HPLA fed | 0.0001 – 0.05 | 0 – 8%  (water control = 0%) | 250 |
| (B) HPLA injected | 0.0001 – 1.0 | 0 – 30%  (water control = 30%) | 100 |
