# Supplementary material for: Repurposing the orphan drug nitisinone to control the transmission of African trypanosomiasis
Source: PLoS Biol. 2021 Jan 26;19(1):e3000796. doi: 10.1371/journal.pbio.3000796 (PMC7837477; doi:10.1371/journal.pbio.3000796)
Supplement: S2 Table — Summary of in vitro NTBC degradation after an hour incubation with insect microsomal preparations and recombinant CYP6P3 in the presence of NADPH. (DOCX) [file pbio.3000796.s003.docx]

**S2 Table**. The HPLC depletion assay was used to determine if NTBC is metabolised by *Glossina* P450 enzymes (detoxification enzymes). Summary of *in vitro* NTBC degradation after an hour incubation with insect microsomal preparations and recombinant CYP6P3 in the presence of NADPH.

| **Microsomes/P450**  **membranes** | **Percentage of NTBC Depleted^a^** | **Replicates** | **± STDEV^b^** |
| --- | --- | --- | --- |
| ***Glossina morsitans*** | 0 | 3 | N/A |
| ***Aedes aegypti*** | 0 | 3 | N/A |
| ***Anopheles gambiae*** | 0 | 3 | N/A |
| **CYP6P3** | 0 | 3 | N/A |
| **CYP6P3+b5^c^** | 10.9 | 3 | 5.4 |

**^a^**NTBC depletion calculated from the chromatographic separation of the drug at retention time (5.2 minutes) separated from the enzyme mixture and incubated for one hour at 30^o^C in the presence of 0.5 mM NADPH, relative to the NTCB peak area in the absence of NADPH, **^b^**standard deviation and **^c^**positive control for activated microsomes.
