# Supplementary material for: Repurposing the orphan drug nitisinone to control the transmission of African trypanosomiasis
Source: PLoS Biol. 2021 Jan 26;19(1):e3000796. doi: 10.1371/journal.pbio.3000796 (PMC7837477; doi:10.1371/journal.pbio.3000796)
Supplement: S3 Table — NTBC stock solutions were stored in either opaque (dark) tubes to protect from light exposure or in translucent tubes (light). Tube storage was either at room temperature (RT; 25°C) or refrigerated (4°C) over a 5-week time period. (DOCX) [file pbio.3000796.s004.docx]

**S3 Table.** NTBC stability under different environmental conditions (light exposure and storage temperature). NTBC stock solutions were stored in either opaque (dark) tubes to protect from light exposure or in translucent tubes (light). Tube storage was either at room temperature (RT; 25°C) or refrigerated (4°C) over a 5-week time period.

| **NTBC final concentration**  **(mg/ml)** | **Stock NTBC**  **(0.01 mg/ml)** | **Horse blood**  **(diluent)** | **% tsetse mortality**  **(bioassay)** |
| --- | --- | --- | --- |
| **0.001 mg/ml (dark-RT)** | 100 µl | 900 µl | 100 |
| **0.001 mg/ml (light-RT)** | 100 µl | 900 µl | 100 |
| **Sucrose 10% (RT)^1^** | 100 µl | 900 µl | 0 |
| **0.001 mg/ml (dark-4C)** | 100 µl | 900 µl | 100 |
| **0.001 mg/ml (light-4C)** | 100 µl | 900 µl | 100 |
| **NTBC control 0.001 mg/ml^2^** | 100 µl | 900 µl | 100 |
| **Sucrose 10% (4C)^1^** | 100 µl | 900 µl | 0 |

**^1^** NTBC-free, negative controls (green) were stored at ambient insectary conditions (**RT**) or at 4°C in the fridge (**4C**). **^2^** Positive controls were bloodmeals supplemented with a lethal concentration (0.001 mg/ml) of fresh NTBC.
