# Supplementary figures and images for: Repurposing the orphan drug nitisinone to control the transmission of African trypanosomiasis
Source: PLoS Biol. 2021 Jan 26;19(1):e3000796. doi: 10.1371/journal.pbio.3000796 (PMC7837477; doi:10.1371/journal.pbio.3000796)

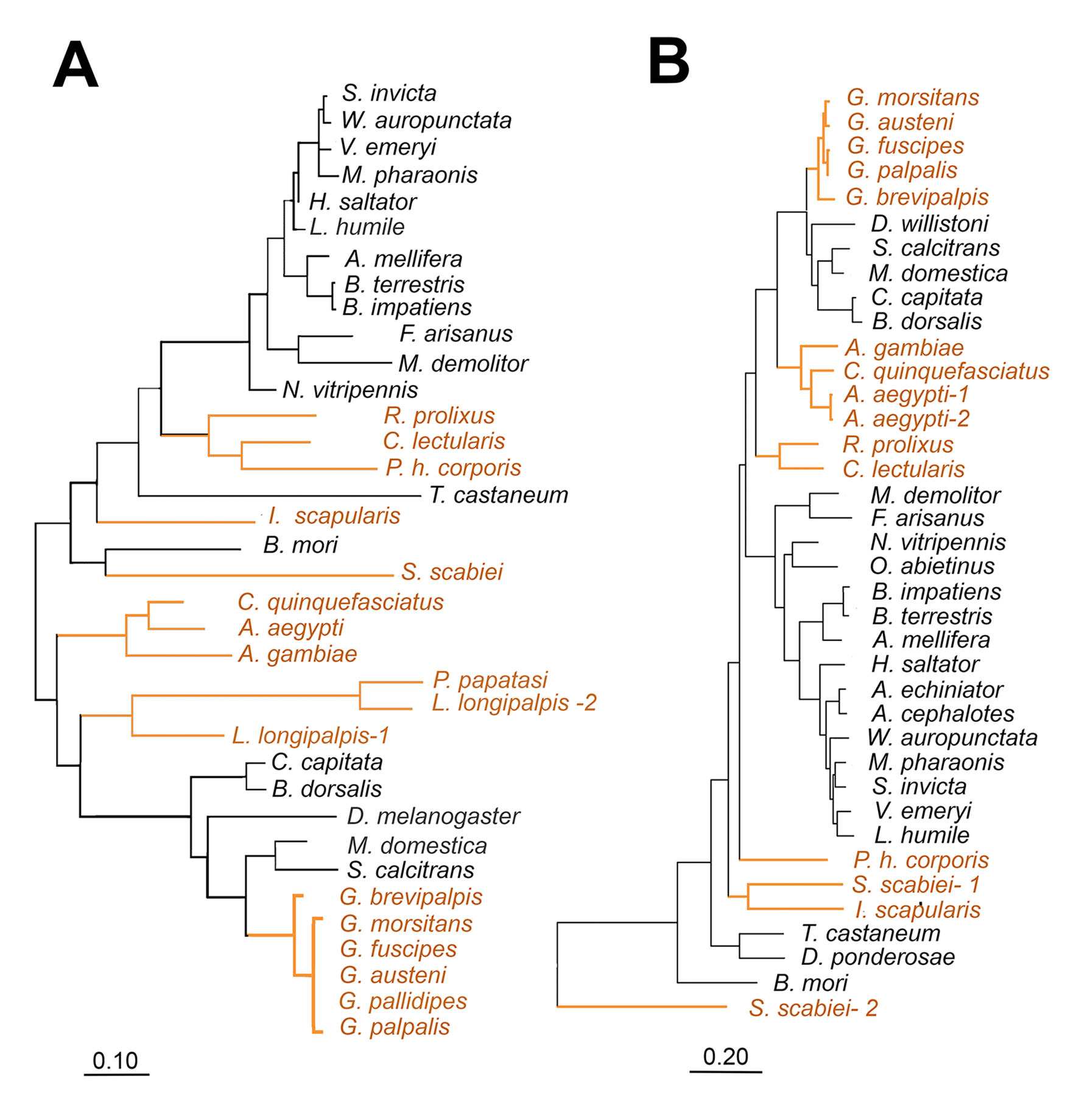

Supplement: S1 Fig — ML phylogenetic trees were created from full-length protein sequences of TAT (A) and HPPD (B) from several insect species. The species and branches highlighted in orange indicate blood-feeding insects. A partial sequence coding for HPPD was identified for G. pallidipes, but it was excluded from the analysis because it was not complete. Scale bars (branch lengths) correspond to the mean number of amino acid substitutions per site on the respective branch. HPPD, 4-hydroxyphenylpyruvate dioxygenase; ML, maximum likelihood; TAT, tyrosine aminotransferase. (TIF) [file pbio.3000796.s005.tif]

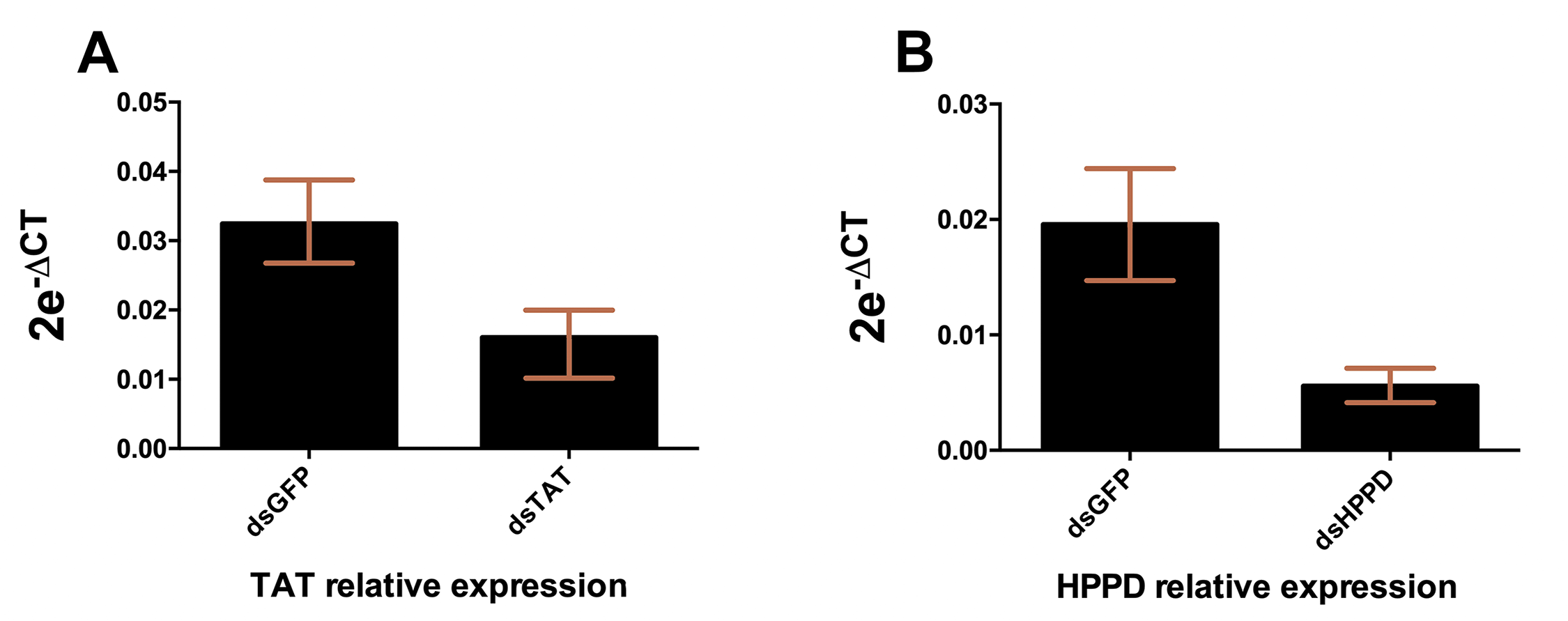

Supplement: S2 Fig — (A) TAT (unpaired t test with equal SD. N = 4–5 p = 0.05) knockdown. (B) HPPD knockdown (unpaired t test with equal SD. N = 5–4 p = 0.04). Data are shown as mean ± SEM. dsRNA, double-stranded RNA; HPPD, 4-hydroxyphenylpyruvate dioxygenase; TAT, tyrosine aminotransferase. (TIF) [file pbio.3000796.s006.tif]

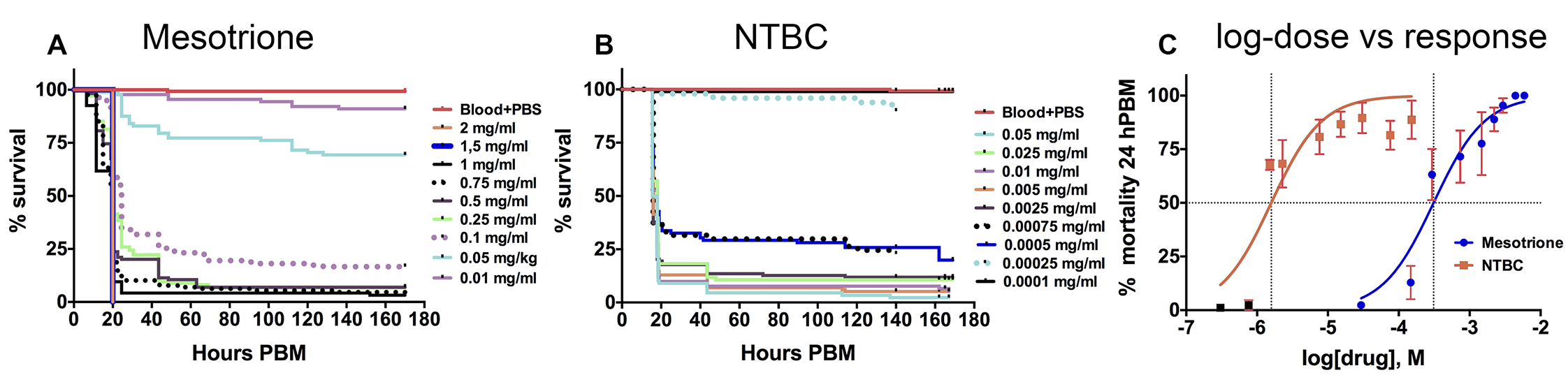

Supplement: S3 Fig — The survival of tsetse after feeding on blood supplemented with either mesotrione (A) or NTBC (B). Dose-response curves were calculated at 24 hours after the bloodmeal (C). Six independent experiments were performed (n = 16–28 insects per dose). Total number of tsetse used to generate the dose-response curves were n = 1,018 for mesotrione and n = 914 for NTBC. Data are shown as mean ± SEM. NTBC, nitisinone. (TIF) [file pbio.3000796.s007.tif]

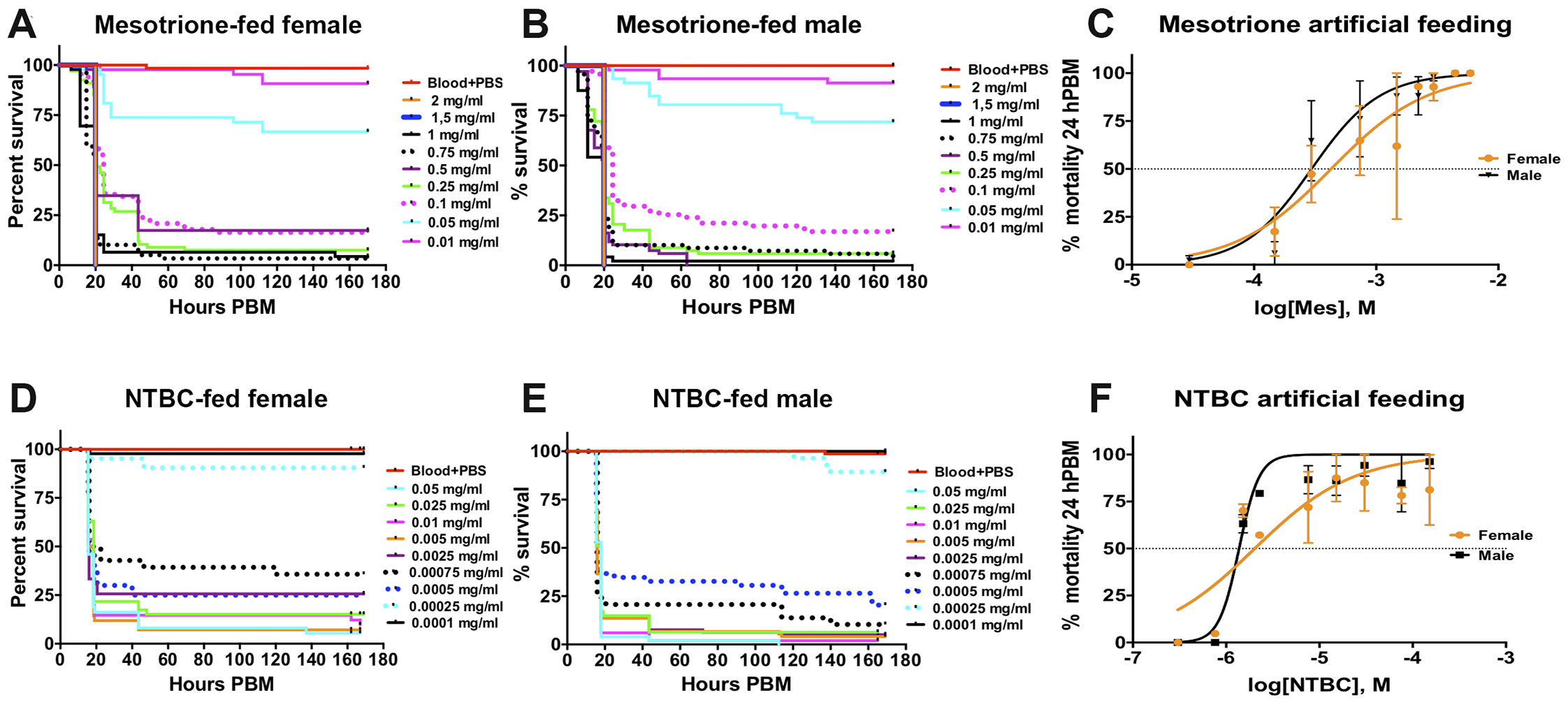

Supplement: S4 Fig — (A) Mesotrione-fed female tsetse (total insects: 483; LD50: 427.5 μM; 95% CI: 189.5–737.4) and (B) Mesotrione-fed male tsetse (total insects: 536; LD50: 304.9 μM; 95% CI: 133.3–507.8). (C) Dose-response curves calculated 24 hours after mesotrione feeding. Data are shown as mean ± SEM. (D) NTBC-fed female tsetse (total insects: 403; LD50: 2.8 μM; 95% CI: 0.38–8.5). (E) NTBC-fed male tsetse (total insects: 511; LD50: 1.7 μM; 95% CI: 0.24–4.32). (F) Dose-response curves calculated 24 hours after NTBC feeding. Three independent experiments were performed, each with n = 26–30 tsetse per dose. Data are shown as mean ± SEM. HPPD, 4-hydroxyphenylpyruvate dioxygenase; NTBC, nitisinone. (TIF) [file pbio.3000796.s008.tif]

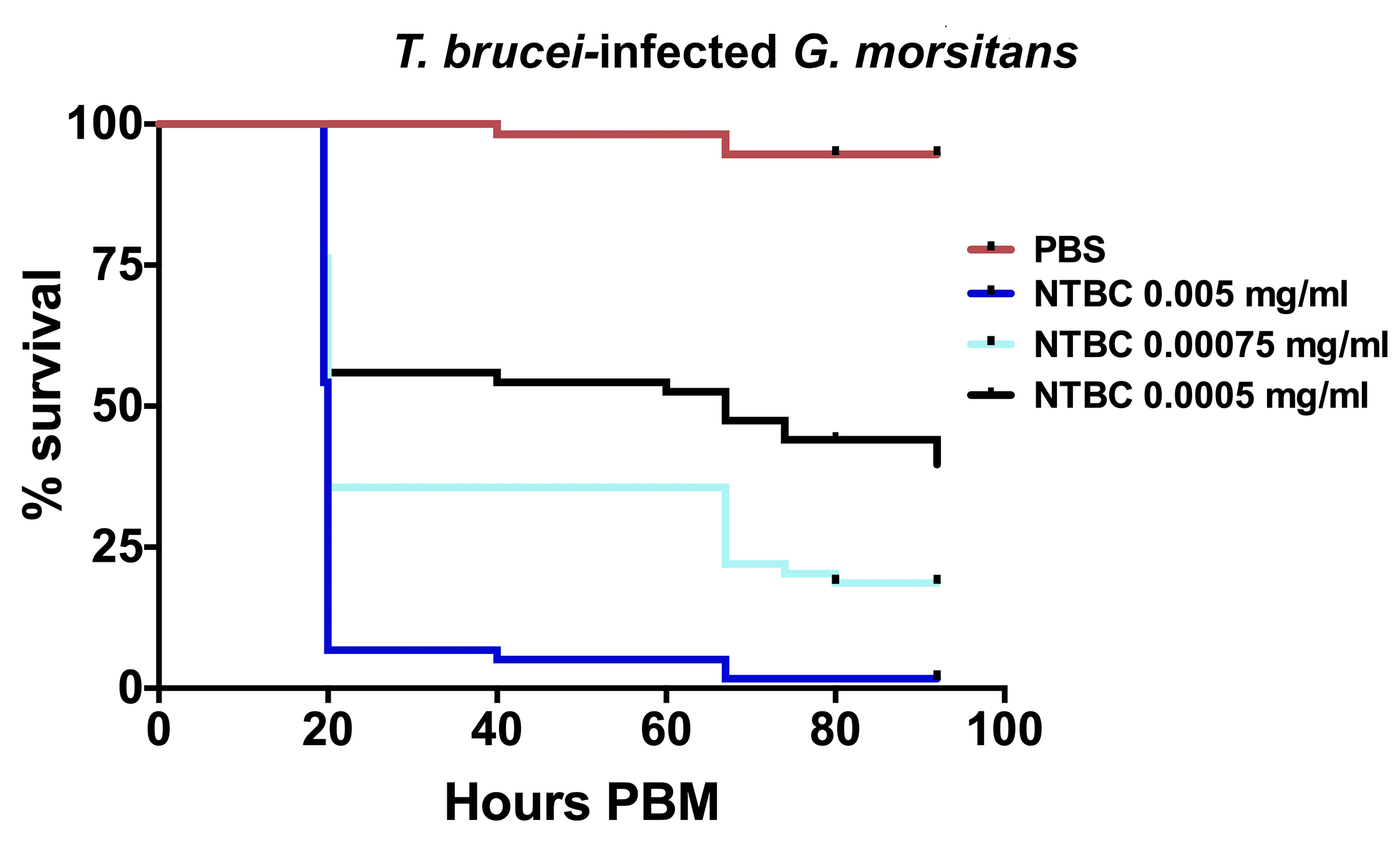

Supplement: S5 Fig — Two independent experiments were performed, each with n = 26–30 tsetse per dose (233 insects in total). NTBC, nitisinone; PBS, phosphate-buffered saline. (TIF) [file pbio.3000796.s009.tif]

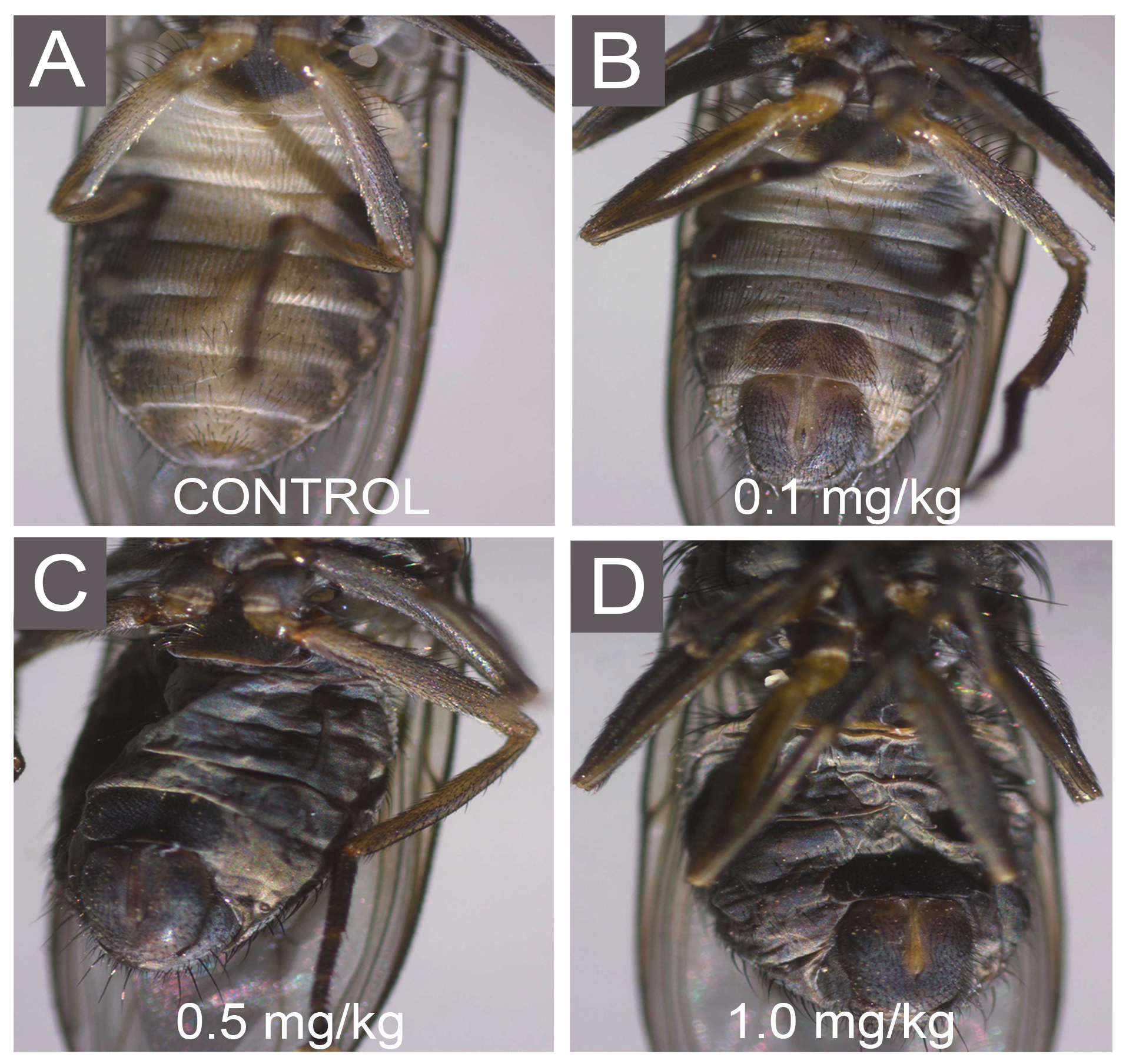

Supplement: S6 Fig — G. pallidipes flies were fed on anaesthetised rats that received an oral dose of sterile PBS (A) or 0.1 mg/kg (B), 0.5 mg/kg (C), or 1 mg/kg NTBC. Survival was recorded for 26 hours post feeding. NTBC, nitisinone; PBS, phosphate-buffered saline. (TIF) [file pbio.3000796.s010.tif]

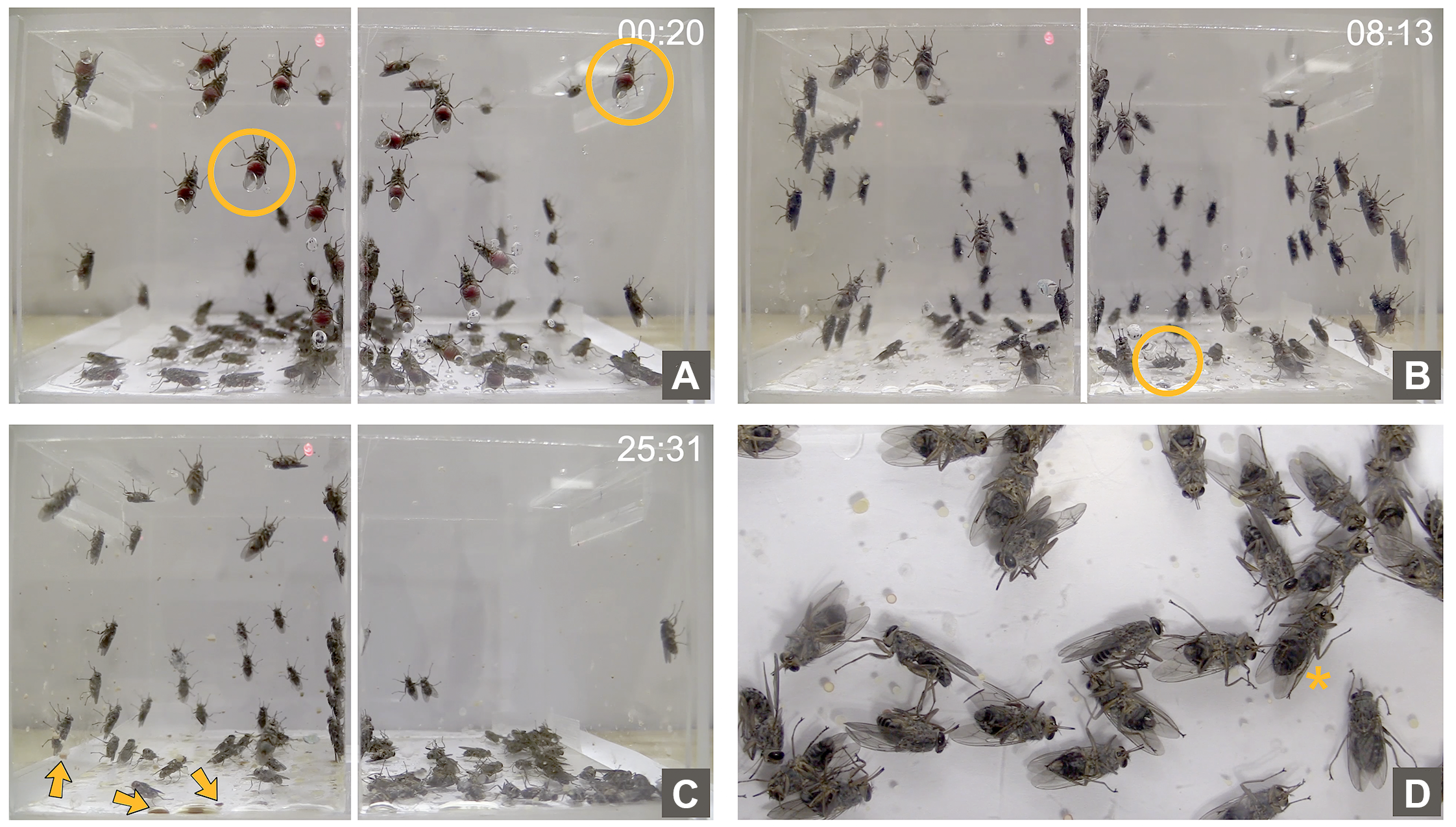

Supplement: S7 Fig — (from S1 Video) Panels (A) to (C) have control (untreated) tsetse on the left and NTBC-treated (0.001 mg/ml) flies on the right. (A) In both groups, the excretion of water within the first 20 minutes after feeding is due to a process called diuresis. (B) The first fly to be partially paralysed (fly on back, yellow circle) in the NTBC-treated group occurs 8 hours after ingesting the bloodmeal. (C) Evidence of bloodmeal digestion in control flies as evidenced by dark excreta (yellow arrows) that is absent in NTBC-treated flies. (D) Top view of NTBC-treated compartment highlights the characteristic NTBC-treated, blackened abdomens (*). All flies are dead (black eyes) or dying (fully paralysed) by 28 hours post ingestion of NTBC. NTBC, nitisinone; PBS, phosphate-buffered saline. (TIF) [file pbio.3000796.s011.tif]

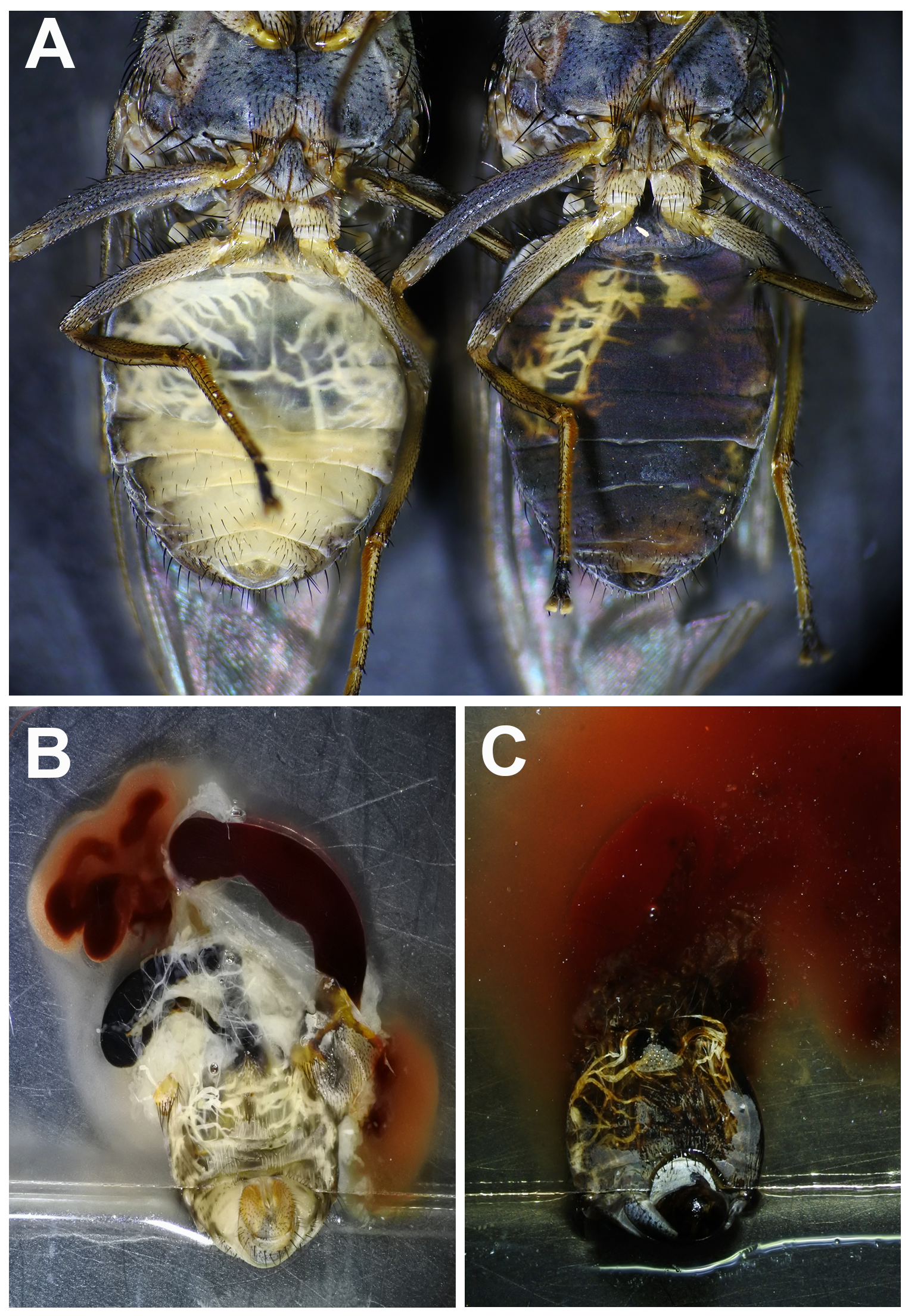

Supplement: S8 Fig — External and internal tissue destruction of NTBC-treated tsetse: (A) tsetse fed regular blood on left and NTBC-treated blood on the right. Internal abdominal liquefaction was demonstrated by placing tsetse abdomens under a glass slide and applying pressure to squeeze out tissues: (B) control fly and (C) NTBC-treated fly. NTBC, nitisinone. (TIF) [file pbio.3000796.s012.tif]

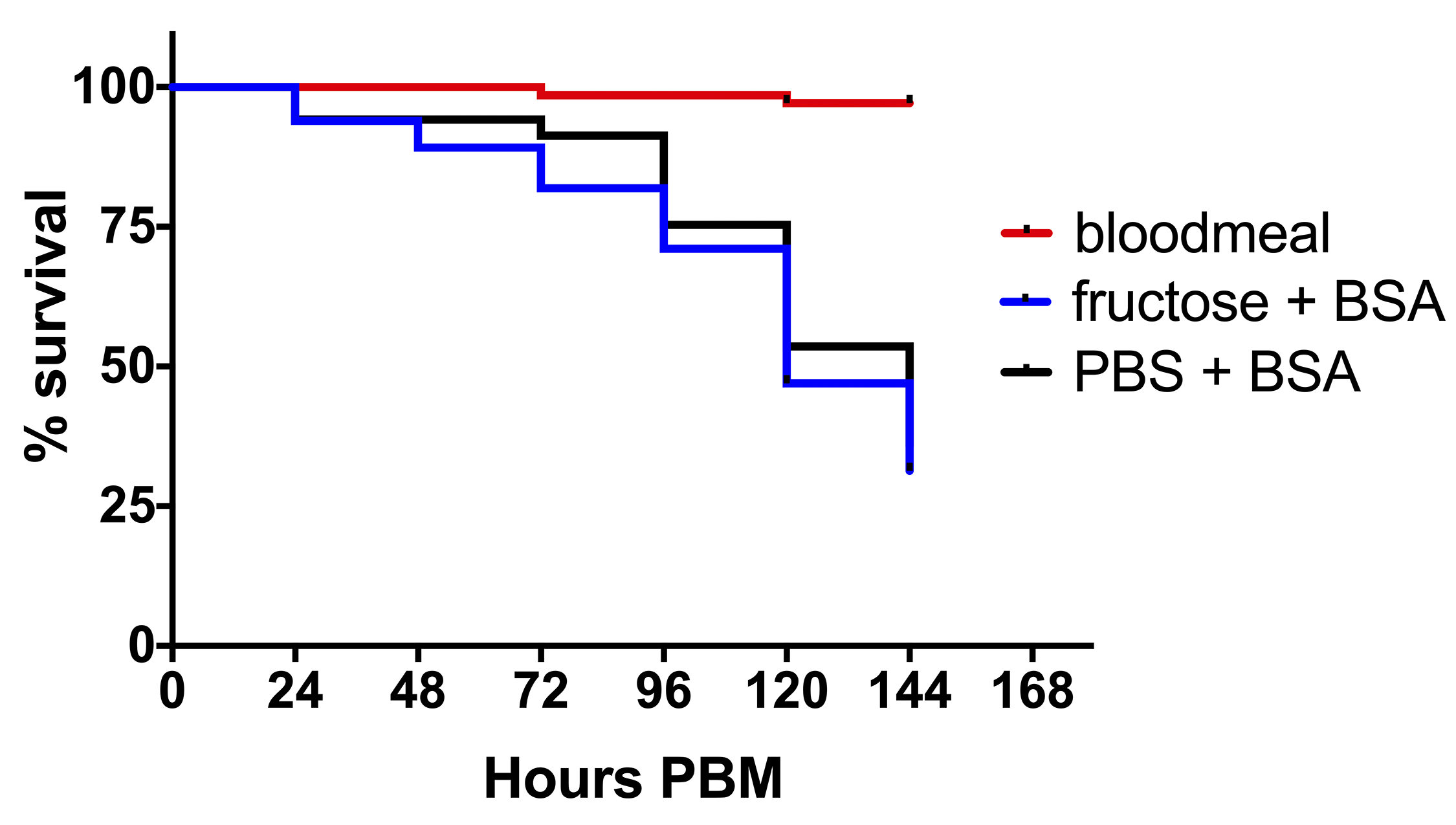

Supplement: S9 Fig — Only a small percentage (approximately 6%) of flies died 24 hours after feeding with either PBS-BSA or fructose-BSA. Moreover, both groups showed a comparable mortality rate until day 6, when they reached approximately 50% of the mortality compared to the group of flies fed only with horse blood. These experiments provide evidence that (1) the 24-hour mortality shown in Fig 4B is due to the addition of NTBC; (2) protein degradation is important for NTBC killing; and (3) tsetse flies do not appear to obtain energy from ingesting 0.1% fructose. Daily feeds were mandatory because the flies are highly susceptible to dehydration as they quickly process the nutritionally poor meal. Fly mortality was daily recorded for a period of six days. Two independent experiments were performed, each with n = 18–51 tsetse per treatment (221 insects in total). BSA, bovine serum albumin; NTBC, nitisinone; PBS, phosphate-buffered saline. (TIF) [file pbio.3000796.s013.tif]

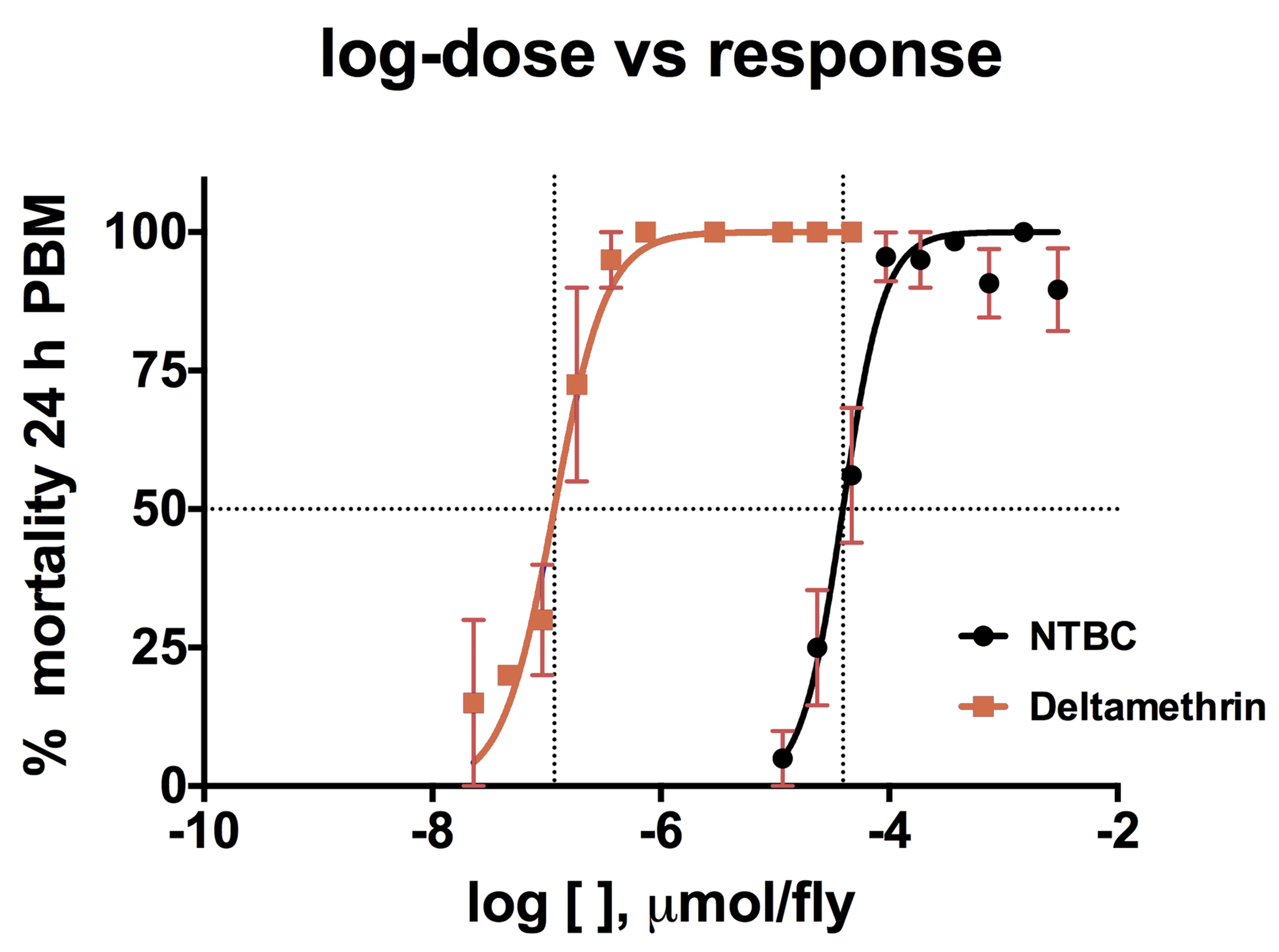

Supplement: S10 Fig — Drugs were applied immediately after tsetse had taken a bloodmeal. Three independent experiments were performed: n = 10–20 insects per dose. The data for the dose-response curves for deltamethrin (n = 339 flies) and NTBC (n = 449 flies) are shown as mean ± SEM. NTBC, nitisinone. (TIF) [file pbio.3000796.s014.tif]

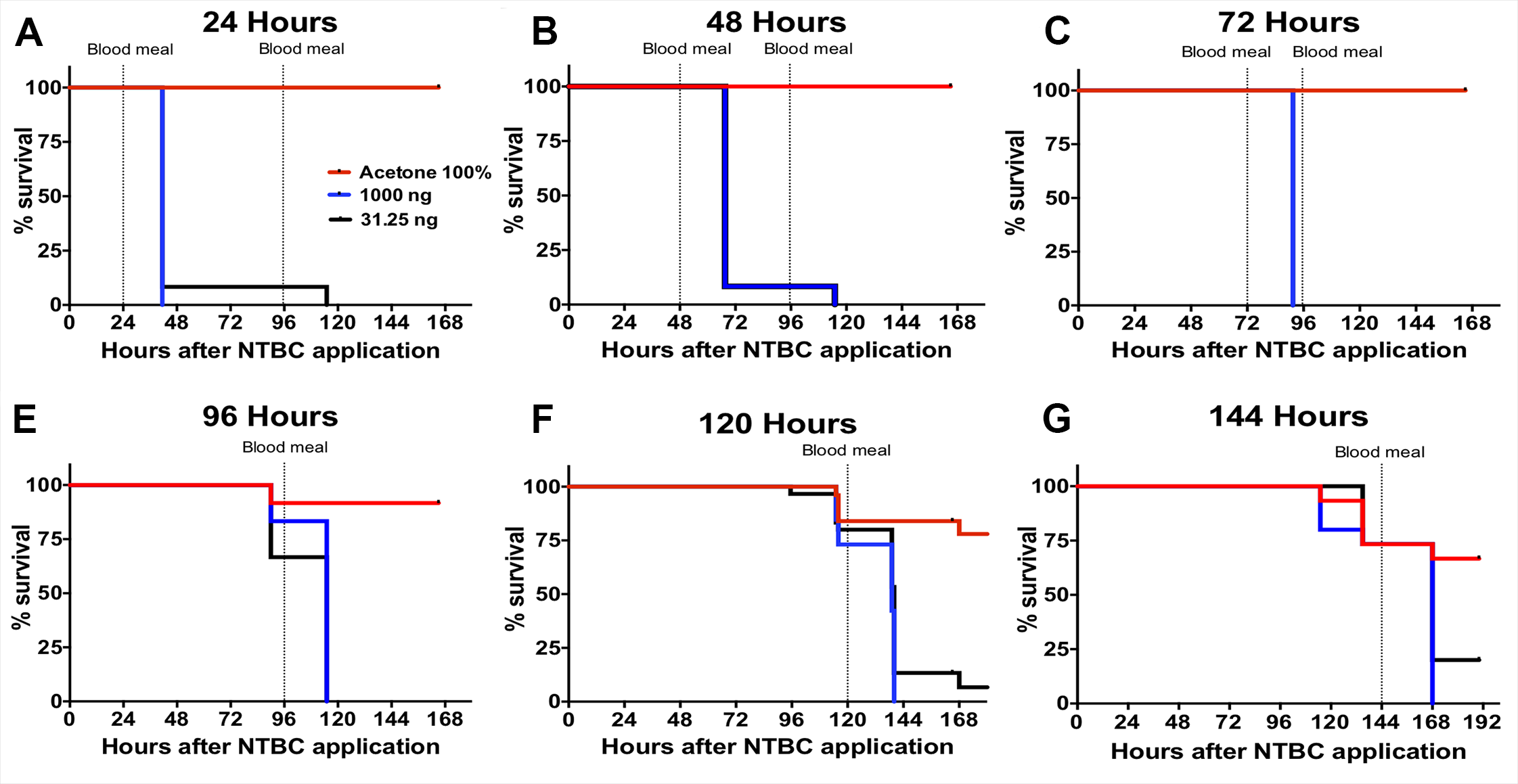

Supplement: S11 Fig — The percent survival of G. m. morsitans after topically applying either acetone (red) or NTBC: 1,000 ng (blue) or 31.25 ng (black). Once the solution was applied, tsetse were offered a bloodmeal every 24 hours to measure the residual activity of absorbed NTBC. Flies were fed at either 24 hours (A), 48 hours (B), 72 hours (C), 96 hours (D), 120 hours (E), or 144 hours (F) after topical application. Vertical dotted lines indicate the timing of each bloodmeal. Two independent experiments were performed, each with n = 10–12 insects (315 insects in total). NTBC, nitisinone. (TIF) [file pbio.3000796.s015.tif]

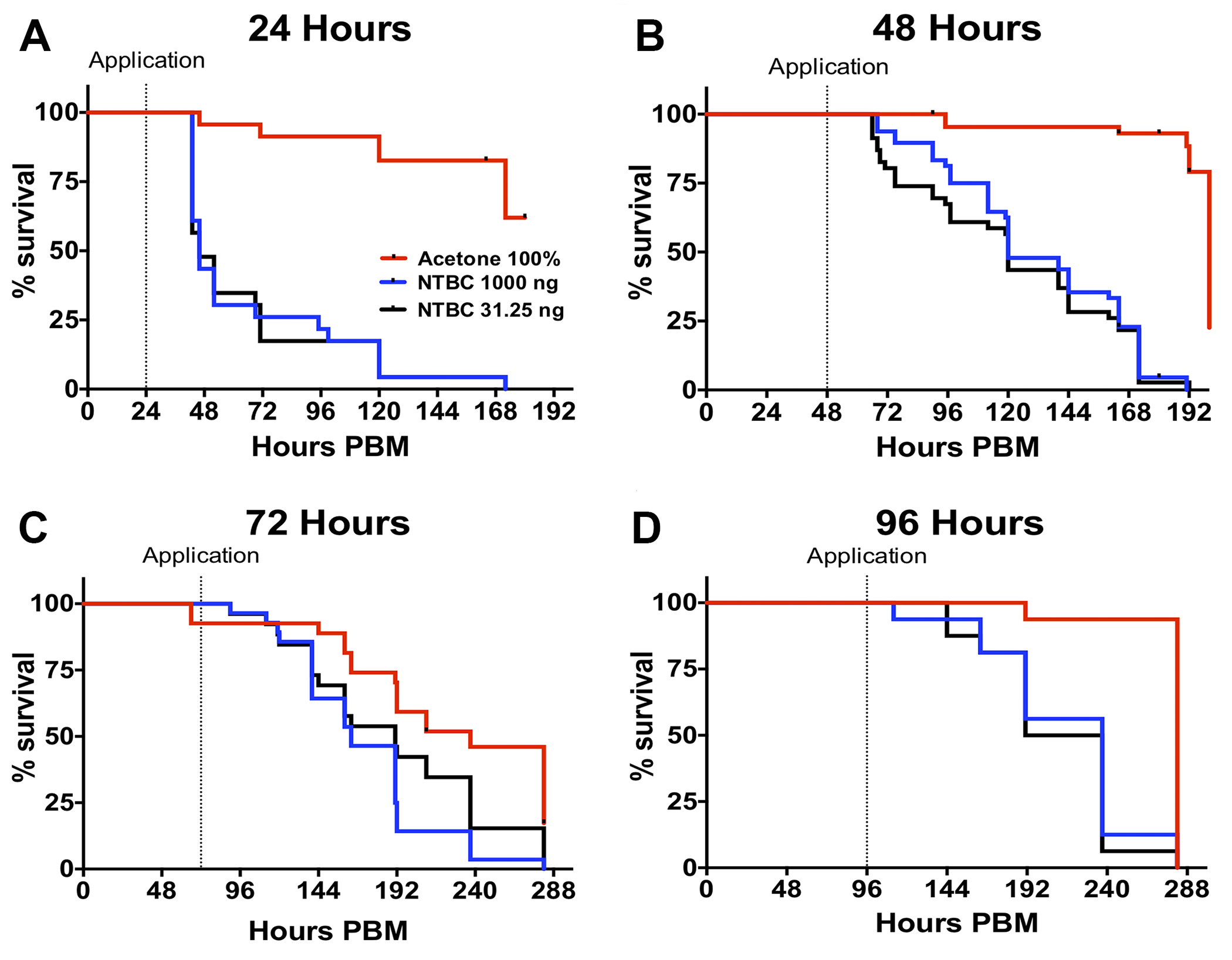

Supplement: S12 Fig — Acetone (red) or NTBC (1,000 ng, blue; 21.25 ng, black) was topically applied at (A) 24 hours, (B) 48 hours, (D) 72 hours, and (D) 96 hours after a fly had ingested a single bloodmeal. Two independent experiments were conducted, each with n = 10–12 insects (306 insects in total). NTBC, nitisinone; PBM, post-blood meal. (TIF) [file pbio.3000796.s016.tif]

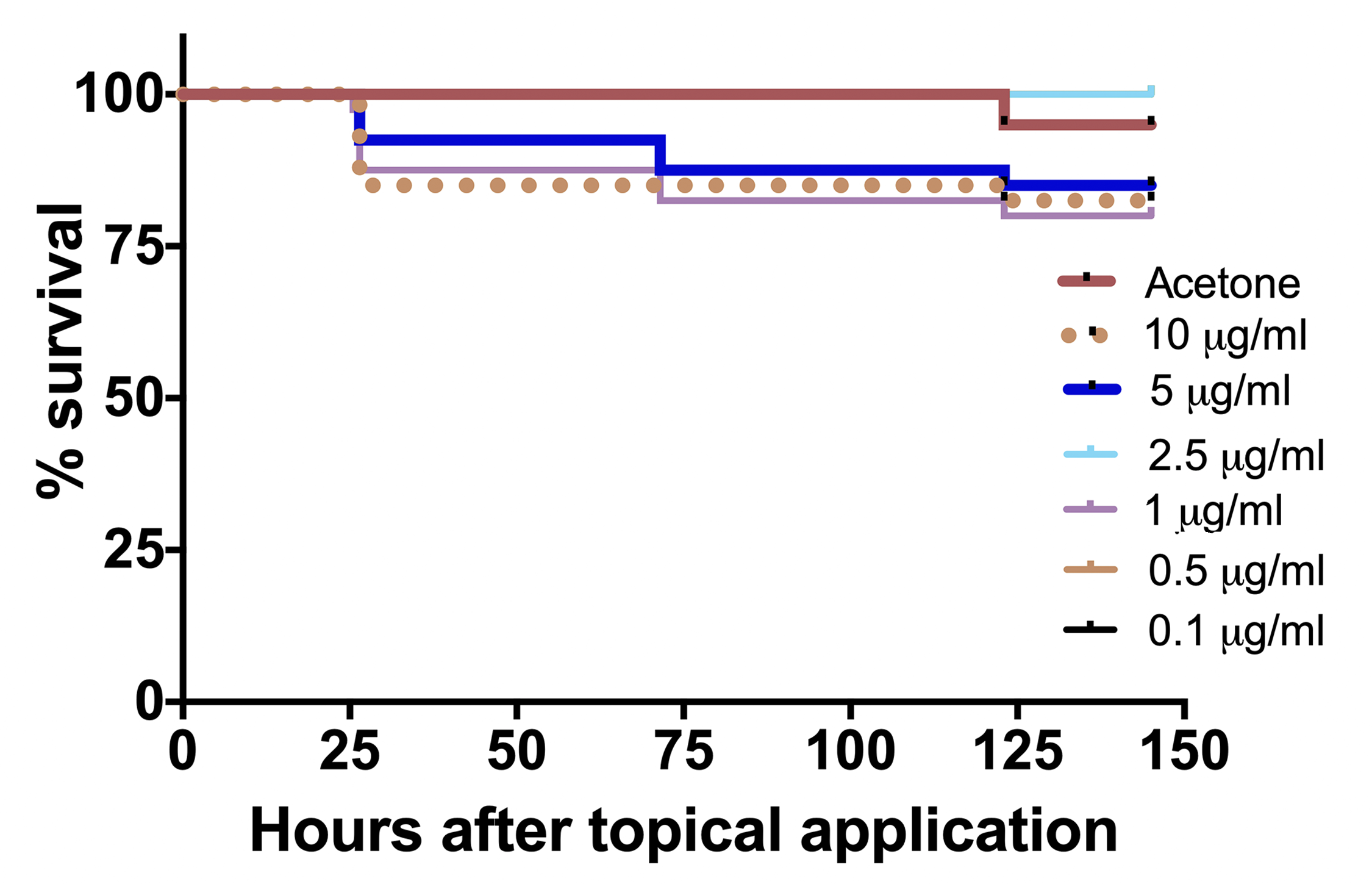

Supplement: S13 Fig — Two independent experiments were performed, each with n = 10–30 flies per dose tested (190 insects in total). (TIF) [file pbio.3000796.s017.tif]

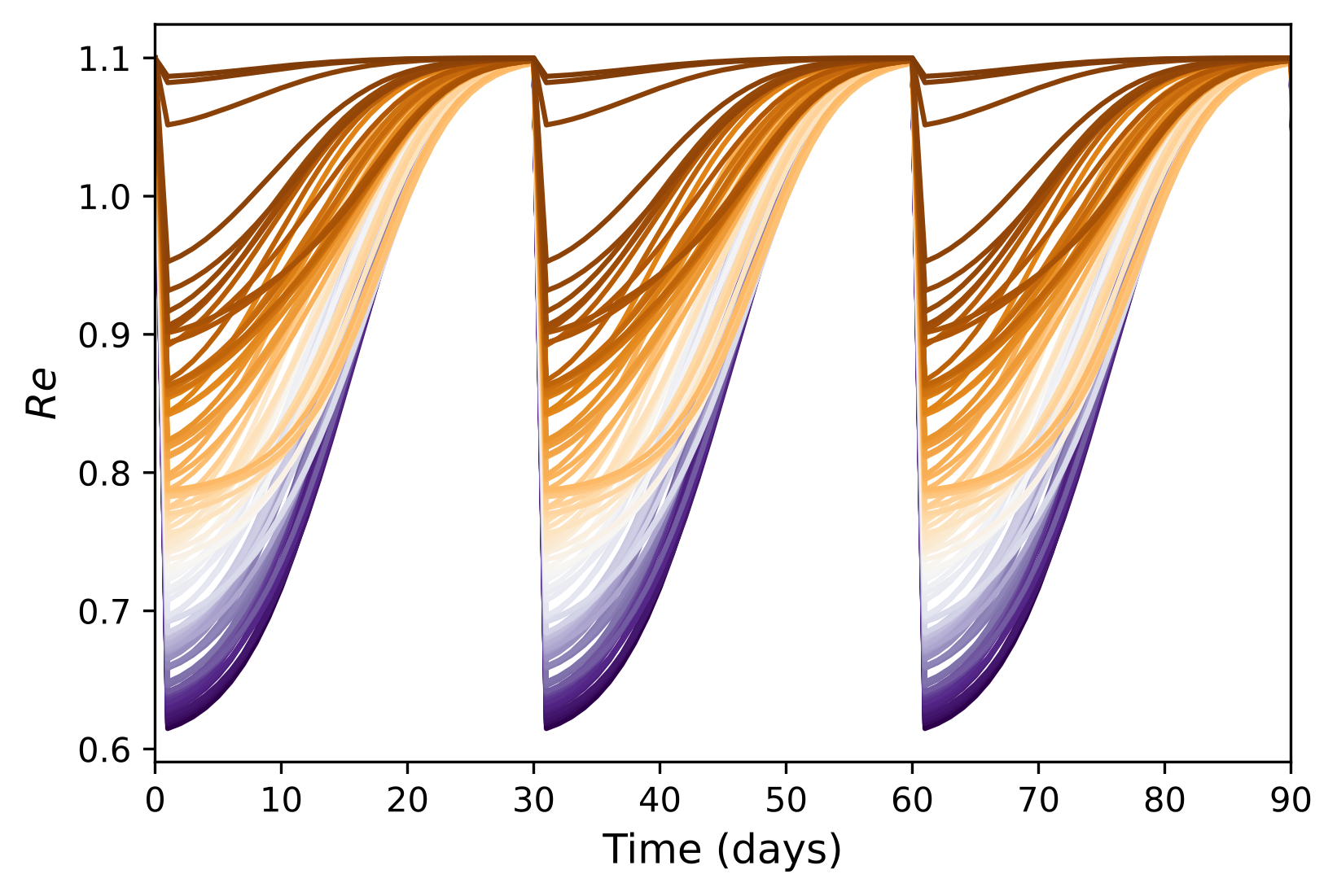

Supplement: S14 Fig — The different lines correspond with randomly selected distributions of bites among humans, livestock, and wildlife. Where wildlife make up the large majority of tsetse bloodmeals, only limited control is achievable (dark brown) but where humans and livestock make up the majority of bloodmeals, good levels of control can be achieved (blue). NTBC, nitisinone. (TIF) [file pbio.3000796.s018.tif]

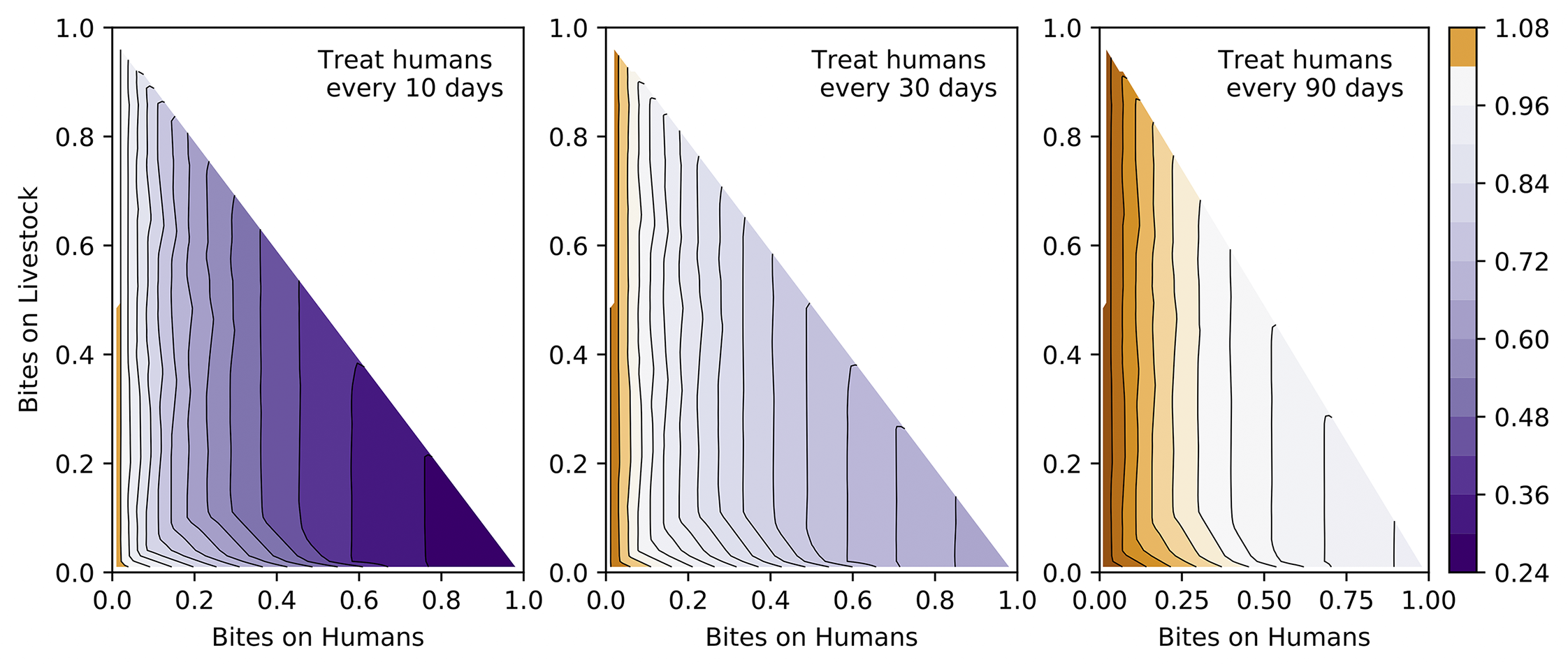

Supplement: S15 Fig — Treatments are conducted either every 10 days (left column), every 30 days (middle column), or every 90 days (right column). The axes denote the proportional split of bloodmeals ingested by local tsetse vectors between humans, livestock, and wildlife (where proportion of bites on wildlife are 1 - (bites on humans + bites on livestock)). NTBC, nitisinone. (TIF) [file pbio.3000796.s019.tif]
