# Supplementary material for: Repurposing the orphan drug nitisinone to control the transmission of African trypanosomiasis
Source: PLoS Biol. 2021 Jan 26;19(1):e3000796. doi: 10.1371/journal.pbio.3000796 (PMC7837477; doi:10.1371/journal.pbio.3000796)
Supplement: Attachment — Submitted filename: Sterkel et al Point by point response.pdf [file pbio.3000796.s022.pdf]

We would like to thank all reviewers for the constructive comments and suggestions.

Reviewer #1:

- *This is a well-conducted and exciting study on the potential of the FDA-approved drug nitisinone to control populations of hematophagous vectors. The body of evidence provided in the paper convincingly demonstrates that nitisinone (a tyrosine degradation inhibitor) can kill tsetse flies upon blood feeding by promoting toxic accumulation of tyrosine. The potency of the drug at concentrations that are compatible with mass drug administration in the human population, the versatile application (oral or topical) and the environmental friendliness (harmless to bumble-bees) make nitisinone a very promising candidate for drug-based vector control. The paper is clearly written, and I only have minor suggestions to improve it, listed below in decreasing order of importance.*

  Response: Many thanks for your positive comments.

- *It would be interesting to discuss the likelihood that flies (or other insects) could evolve nitisinone resistance by other means than metabolic detoxification (e.g., target insensitivity). In the dose-response experiments, a small proportion (10-15%) of flies seems to survive even at high drug concentration (e.g., Fig. 1d). This observation is consistent with the existence of some genetic variation and/or phenotypic plasticity in nitisinone tolerance.*

  Response: We have now included two paragraphs in the Discussion section related to these two issues. First (page 15, lines 355-368), newly emerged tsetse flies can ingest bloodmeals of different sizes. We removed all flies that did not have red abdomens, however the quantity of blood ingested is difficult to estimate as a fly begins to remove water (diuresis) within 10 minutes of feeding. It is likely that the small proportion of flies that survived the high drug concentrations ingested a smaller bloodmeal, and as a result, a smaller drug dose. This issue is certainly more pronounced in younger flies.

  Second, regarding nitisinone tolerance (page 17, lines 408-431), we think mutations that would affect the binding of NTBC to HPPD may also alter the affinity for HPPA. Thus, due to the importance of HPPD in the physiology of hematophagous arthropods, mutations that reduce its affinity for HPPA would probably be lethal. However, we cannot rule out the existence of some genetic variation or phenotypic plasticity that could confer tolerance to hematophagous vectors towards HPPD inhibitors. Certainly, after >20 years of using common agriculture-based HPPD inhibitors like mesotrione, resistant weeds have emerged but interestingly no target-site mutations of the HPPD genes has been associated with post-emergence resistance. Moreover, no gene duplication or over-expression of HPPD, before or after herbicide treatment, was detected. In addition, a new gene called HIS1 (HPPD Inhibitor Sensitive 1) has been linked with resistance to several triketone herbicides (including mesotrione) in rice plants, but our Vectorbase search (www.vectorbase.org) did not find orthologous genes in the genomes of several insects, thus suggesting the absence of this gene in invertebrates.

- *Since PLoS Biology has no restrictions on the number of figures, some of the data relegated to the supporting information could be included in the main body of the paper. For instance, Figs. S6, S7 and S8 could be combined and provided as an additional main figure.*

  Response: As suggested, we have included a new figure (Fig. 4) in the main part of the manuscript, which is composed of former figures S7 and S8, and now shows the potency of HPPD inhibitors in serum and BSA. Fig S6 was excluded from the manuscript (see below). The mathematical model is now Fig. 5. The number of figures in the supplemental material have also been changed accordingly.

- *It is not expected that the authors carry out additional experiments with mosquitoes, but perhaps the collateral effects of nitisinone on mosquito vector species (if they are considered plausible) could be mentioned in the discussion as an additional benefit to the proposed strategy.*

  Response: We have included a short comment at the end of the Discussion section on the additional benefits that a NTBC-based strategy may bring for the control of other vector-borne diseases (page 18, lines 432-442). In addition, the Oliveira lab has a manuscript currently under revision in a peer-reviewed journal (bioRxiv doi.org/10.1101/669747) on the use of HPPD inhibitors for mosquito control, and the Acosta-Serrano group is currently drafting another manuscript on use of NTBC as endectocide for malaria control.

- *Some guidance in the caption could help the unfamiliar reader to navigate the ternary plots shown in Fig. 4. For example, placing the labels at the corners (not the edges) would make it easier to interpret the simulation results. Frequency (in days) should probably refer to time interval between applications.*

  Response: We apologise for how unclear this figure was – we have replotted our simulation outputs in what we hope is a clearer format. The original message from the modelling has not changed; i.e. regular treatments of NTBC used as an endectocide at high coverage levels are needed to control disease transmission. Since a new figure has been added (explained above), the model is now Fig. 5.

- *Line 108: why are the data not shown?*

  Response: We performed feeding experiments with insects of both sexes separately. Since we did not observe differences in the time of death nor in the susceptibility to lethal doses, we combined the data obtained from both sexes into single graphs. We have now included a new supplementary figure (Fig. S4) showing the separate mortality data from females and males that were fed NTBC and mesotrione-supplemented blood. Furthermore, the sex of the specimens used in each experiment have been added to the Methods section.

Reviewer #2:

- *The manuscript by Sterkel et al. describes the implementation of a tyrosine catabolism inhibitor (nitisinone - NTBC) as a putative insecticide targeted at control of blood feeding arthropods with tsetse flies being the focus of this work.*

  The manuscript is well written and easy to read, the materials and methods are comprehensive and well described and the data is presented clearly. I feel the manuscript is well suited to PLoS Biology. However, I think some of the statements in the manuscript regarding the data are overinterpreted and either need to be toned down or additional controls/experimental work need to be included to provide support for these statements. I also think the final figure could use some modifications to make it easier to interpret and should include one alternative condition.

  Response: Thank you for your insightful perspective. As suggested, we have toned down the interpretation of our findings (see below).

- *The authors perform a thorough analysis of the pathology resulting from in nitisinone treatment revealing extensive disruption and breakdown of internal tissues/organ systems. They demonstrate via metabolomic analysis that treatment with the inhibitor results in accumulation of toxic levels of tyrosine and hydroxyphenylpyruvic acid as a result of the HPPD inhibition. They go on to examine the potential for excess melanization activity and/or haem accumulation to be the cause of pathology. The authors state that they inhibited phenoloxidase (PO) activity with treatment of phenylthiourea (PTU), however, the experiment lacks a positive control to demonstrate the inhibitor is working. The inclusion of a positive control such as injected beads that trigger the melanization reaction by the immune system in the presence and absence of PTU would make the argument more convincing. In addition, thes PTU treatment seems to have a significant negative impact on the survival of the flies. It would be more convincing if the authors dissected out tissues from PTU and NTBC treated flies in a similar manner to what is shown in Figure 2 A+B to show that the melanization found in the NTBC treated flies alone disappears in the presence of PTU.*

  Response: Thank you for flagging this experiment, which in fact was also highlighted by reviewer #3. We have now repeated these experiments and have expanded them to include evaluation of a range of different NTBC concentrations (please see below Panel A) and PTU concentrations. When flies were fed on bloodmeals containing 0.001 mg/ml NTBC (lethal dose), in the presence or absence of 0.1 mg/ml PTU, the mortality rate seen in both groups was comparable. However, we also dissected some of the treated insects (please see representative image Panel B below) and found that only the group fed with 0.001 mg/ml NTBC had melanized salivary glands after 24 hours post-bloodmeal (PBM). No salivary gland melanisation in the PTU + NTBC-treated group was observed (n = 10). Furthermore, when flies were fed with bloodmeals containing lower NTBC doses (0.0001 and 0.0005 mg/ml), we observed a delay in NTBC-associated killing, but only when 0.1 mg/ml PTU; this phenotype is lost when PTU is diluted to 0.01 mg/ml. The same results were observed in three independent experiments and were observed in both male and female flies. Thus, this data suggests that transient inhibition of phenoloxidase by PTU may cause a slight delay in the mortality induced by sub-lethal NTBC doses. Additionally, when flies were treated with the lowest dose of NTBC (0.0001

mg/ml), those flies co-ingesting PTU showed identical mortality to the PBS control (solid blue vs. dotted blue line). We postulate the delayed mortality and possible protective effect of PTU was previously overshadowed in the original supplemental figure (former S6 Fig) as we only tested a very high dose of NTBC (0.01 mg/ml), which appears to "overwhelm" the effect of PTU.

A. Mortality Rate

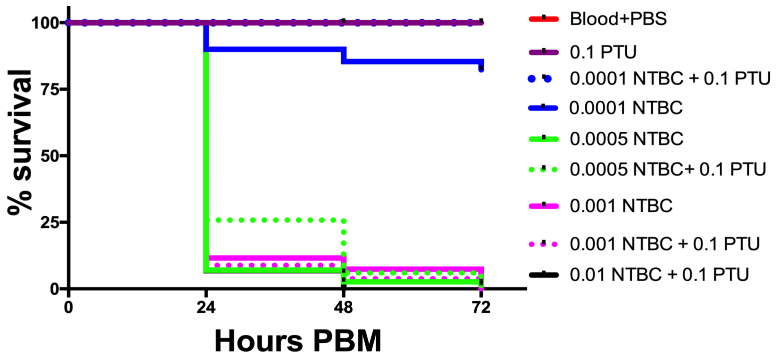

B. Sal. glands melanization (24h PBM)

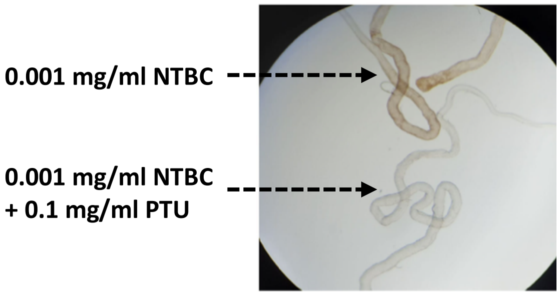

- *Another alternative would be for the authors to mine their metabolomics data to compare the levels of downstream metabolites of the PO cascade such as dopa, dopamine and melanin in the NTBC treated flies.*

  Response: As requested, we looked again into the original metabolomics-MS data, but unfortunately we could not detect metabolites of the phenoloxidase cascade.

  As our recent PTU data above highlights the complexity of *Glossina* PO in the killing induced by NTBC, we have excluded the entire PTU section from the manuscript as it warrants a more in-depth investigation and detracts from the main story of the paper. To understand how tsetse POs contribute to the lethality induced by NTBC, a series of lengthy experiments (difficult to perform given the current circumstances of restricted lab access) are required, which include determining the NTBC susceptibility and tissue analysis of *PO* knockdown flies alongside metabolomics analyses. We are excited by these unexpected findings and feel they are worth investigating in more detail.

- *The authors state that both oral and topical administration of NTBC are effective delivery mechanisms and that dietary protein is essential for its toxicity. In Supplemental figure*

8 they show that the LC50 of NTBC requires ingestion of ~15 mg/ml of protein. This experiment could also use a control group which was fed BSA in the absence of NTBC. Blood is a complex mixture which tsetse have evolved specific mechanisms to deal with, however ingestion of sugar solution infused with BSA has a very different composition/osmolarity to that of blood and may have significant biochemical impacts on the fly. The control group would allow such issues to be accounted for and isolate the effect of protein concentration on NTBC mode of action.

Response: The reviewer has highlighted something important; tsetse are obligate blood feeders, and thus many components of the bloodmeal are crucial for fly survival and fecundity. Although we know flies can survive on BSA-supplemented PBS and/or fructose for a few days, we had never tested for how long they can survive on this diet. As requested, we performed feeding experiments with BSA diluted in either PBS or fructose. We fed the flies daily with either 0.1% fructose-PBS or PBS alone supplemented with 34 mg/ml of BSA. The 34 mg/ml BSA concentration used is based on the published concentration of albumin in horse serum (Barrera et al., 2010). Daily feeds were mandatory because the flies are highly susceptible to dehydration as they quickly process the nutritionally poor meal. Fly mortality was daily recorded for a period of 6 days.

In the figure below we have combined the results from two independent experiments (221 insects in total). An additional group of flies fed with horse blood served as the survival control. Only a small percentage (~6%) of flies died 24 h after feeding with either PBS-BSA or fructose-BSA. Moreover, both groups showed a comparable mortality rate until day 6, when they reached approximately 50% of the mortality compared to the group of flies fed only with horse blood. Upon dissection of a few of the survivors, signs of extreme starvation were evident: collapsed abdomens and emptied fat bodies. In summary, these experiments provide evidence that: i) the 24 h mortality shown in Fig 4B is due to the addition of NTBC; ii) protein degradation is important for NTBC killing; and iii) tsetse flies do not appear to obtain energy from ingesting 0.1% fructose.

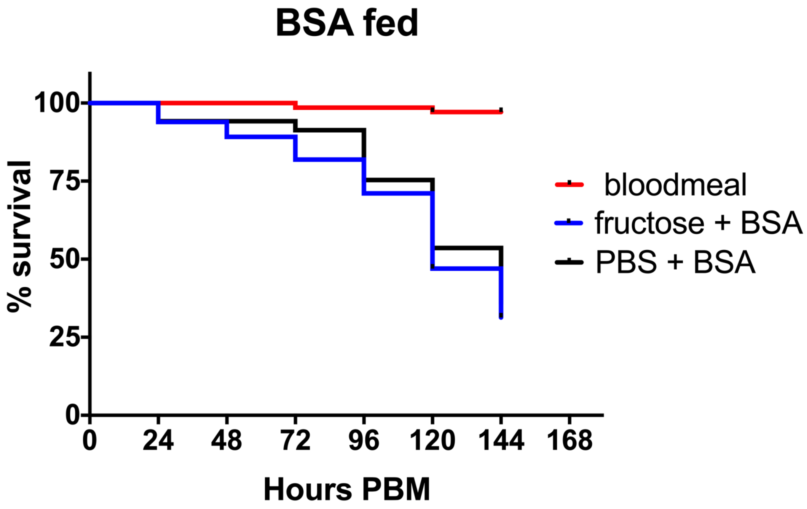

- *As the headline of one of the major sections of the results indicates that "NTBC-associated damage does not depend on phenoloxidase activation or haem, but protein*

concentration in the bloodmeal" I think either this section should be toned down or additional controls should be added to the experiments to bolster this statement.

Response: Agree. As mentioned earlier, the whole PTU section has been withdrawn from the MS; the remaining of this section has been amended accordingly.

- *In the final section which discusses the results of the mathematical modelling of tsetse populations a model is presented which predicts how long NTBC treatment interventions would be required to reduce the population replacement number below 1. I was somewhat confused as to how to interpret the color scale as it would seem that the lower the number of days of treatment required would represent higher efficacy. However, the black indicator at the bottom of the figure indicates that the population is not controlled. The color indicating only 10 days of treatment at the bottom of the gradient is very close to black, so to my eye treatment efficacy is correlating with colour intensity with the exception of completely black dots which are ineffective. If I am interpreting this correctly, I would suggest to reverse the gradient or change the color of the dots that fall into the "Not controlled" category. Another consideration in regards to this figure is that the two panels represent treatment of humans alone and treatment of livestock and humans. Treatment of healthy humans with a drug primarily tested in people with a genetic disease could potentially have unanticipated long term side effects. I think consideration should be given to treatment of livestock alone and that that should be presented as a third condition.*

  Response: We apologize for how unclear this figure was – please find new plots in the updated manuscript (Figure 5) along with this third condition of NTBC distribution (to livestock only), as recommended by the reviewer.

Reviewer #3:

- *The manuscript by M. Sterkel and colleagues describes the effects of genetic and chemical impairment of tyrosine catabolism in two species of Glossinia, the tsetse fly vector of both human and animal African trypanosomiasis, a neglected tropical disease. The authors demonstrate that perturbation of tyrosine catabolism, either through mRNA silencing targeted against tyrosine aminotransferase (TAT) and hydroxyphenylpyruvate dioxygenase (HPPD), or through the use of the selective HPPD inhibitors mesotrione and nitisinone, causes widespread tissue damage, morbidity and mortality in Glossinia spp. following a vertebrate blood meal, or digestion of protein. The authors go on to characterize this effect in detail, demonstrating the insecticidal effects of HPPD inhibitors when included in sugar or blood meals, or through direct external application. The authors conclude that HPPD inhibitors could be used as endectocides in mass drug administration (MDA), livestock treatment, or as an active ingredient in tsetse-targeting interventions. New tools to control tsetse flies are needed, and the presented data are an encouraging step forward.*

  The authors should be commended for presenting a large amount of work on an important subject. However, there are a number of important issues that reduce the relevance of this study.

This study follows a previous publication from some of the same authors detailing similar effects of TAT and HPPD RNAi, and mesotrione and nitisinone on the Trypanosoma cruzi vector Rhodnius prolixus, and the mosquito vector Aedes aegypti. While the present manuscript goes into greater detail on the effects of perturbation of tyrosine catabolism in tsetse, the prior, similar findings in other hematophagic insects strongly reduces the novelty and impact of the present work.

<u>Response</u>: Thank you for the comments and highlighting the sections requiring more clarity and explanation. In previous work (Sterkel et al., 2016), which mainly used *Rhodnius prolixus* as model blood feeder insect, we presented the proof of principle that the inhibition of tyrosine catabolism was lethal to hematophagous arthropods after blood feeding. We did not investigate the molecular mechanisms (metabolomics) underlying the lethal phenotype or model this the potential of this intervention for disease control. Furthermore, our work on *Rhodnius* differs substantially from *Glossina* in that HPPD inhibitors have an immediate effect in *Glossina*, probably reflecting on the different digestion rates between these two insect species. Furthermore, in the current manuscript, we also consider how these inhibitors could be used in a field setting and specifically assessed the mortality on a dipteran pollinator species. Overall, we think the information presented in this manuscript complements our previous publications and provides novel and relevant data that further highlights how HPPD inhibitors may be incorporated into new tools to control tsetse-transmitted African trypanosomiasis and other vector-borne diseases.

- *Throughout, revisions are needed in the presentation of the data. The authors leave the reader to do much of the work to synthesize the data as the results section is, in general, too vague throughout (see specific comments). Data, including dosage concentrations, diluents, controls, median time-to-death, percent mortality at 24 h, should be presented clearly in the results section as well as the method text and not left to the figures. The same is true for figure legends, which lack detail.*

  <u>Response</u>: Please, see specific responses below.

- *Figures are difficult to follow, with very similar looking panels presenting different treatment conditions. All figures should have treatment conditions specified either on top of the graphs or on the x axis.*

  <u>Response</u>: We apologize for the lack of clarity of some of the figures. Most of the revised figures now contain a short description embedded in the figure.

- *Line 195. Although the addition of phenylthiourea may indicate that PO activation is not required for NTBC activity, those experiments do not prove this is the case as the authors do not determine whether PO activity was inhibited in those conditions. So those conclusions aren't warranted.*

  <u>Response</u>: Agree. Please see our previous response to rev #2 regarding the interpretation of our PTU experiments. We have decided to withdraw this section from the paper.

- *Line 278. No details of the model used are provided in the results. Also, the description of those results is minimal. What's the possible impact of the two control measures, and at what coverage level is an impact achieved?*

  The authors should also discuss the feasibility of achieving such high coverage levels as those indicated by the model figure.

  Response: We have updated the way that the model results are presented and included further details of the model in the supporting materials to ensure results can be replicated. Previously, we assumed 100% coverage and following the recommendation of Rev #4 we have reduced this to a more feasible 80% coverage. Importantly, we have changed the tone of the modelling results to emphasize in the Discussion section (lines 317-319) that NTBC is unlikely to comprise a standalone vector control strategy for trypanosomiasis control.

- *'Data not shown' should be presented in supplementary figures.*

  Response: We performed experiments with both sexes of tsetse. Since we did not observe differences in the time of death or in the lethal doses, we combined the data from both sexes onto the same graph. All artificial feeding experiments were performed with each sex fed separately (to ensure a higher feeding rate). Because you have pointed out that this is confusing, we excluded the phrase "Data not shown" from the manuscript and have incorporated the sex used in each experiment in the method section. To provide further result granularity, we have created supplemental Fig S4 to show how male and female tsetse specifically respond to NTBC- or mesotrione-supplemented bloodmeals.

- *The use of "spiked" to describe solutions containing treatment compounds should be avoided. Instead include total makeup of provided solution (e.g. defibrinated horse blood with 10 µM mesotrione). In general, the range, or specific concentrations used in experiments should be stated in the results.*

  Response: The word "spiked" has been replaced for "supplemented with" throughout the manuscript.

- *Line 102-104. State degree of mortality, and knockdown efficiency, for each treatment. Presumably the explanation for incomplete mortality is due to incomplete knockdown, but this is not stated in the text.*

  Response: The following was added to the text (lines 99-100): "However, 100% mortality was not observed, which may be explained by incomplete gene knockdown".

- *Line 121: please provide specific safety information for nitisinone/NTBC.*

  Response: The safety information and adverse effects reported for NTBC are reviewed in reference 19: Lock E, Ranganath LR, Timmis O. (2014). The Role of Nitisinone in Tyrosine Pathway Disorders. Curr Rheumatol Rep. 16: 1–8. doi:10.1007/s11926-014-

0457-0. We also added a new reference 20: European Medicines Agency-Orfadin, INN-Nitisinone. (2004). 1: 1–102 that is available at https://www.ema.europa.eu/en/documents/scientific-discussion/orfadin-epar-scientific-discussion_en.pdf

This document from The European Medicines Agency contains all the information related to the commercially available product information for human use (Nitisinone: Orfadin)

- *Related: nitisinone should be defined once as NTBC at first use, then referred to as that only for the remainder of the text. Currently, references to this drug are inconsistent.*

  Response: The manuscript and the supplementary information section have been modified according to reviewer's suggestion.

- *Line 126: missing reference for standard oral dose of 1 mg/kg nitisinone.*

  Response: The reference (Hall et al., 2001) has been added to the revised MS.

- *Line 129 to 131: There is an extensive literature on both veterinary and human endectocides that should be referenced here.*

  Response: As requested, new references (21-23) covering veterinary and human endectocides have been added.

- *Line 132-134: These data should be described in more detail.*

  Response: A short statement on the efficacy of NTBC killing in trypanosome-infected flies has been added (lns 128-131). A description on how this experiment was completed is further described in the Methods section (lns 592-597).

- *Line 136-143: Again the data should be described fully - median-time-to-death etc - including the fact that mortality was incomplete, and (as stated in the discussion, but not here) that surviving flies suffered some morbidity effects*

  Response: In this experiment, the survival was only been recorded for 26 hours. The phenotype observed matched the artificial feeding experiments, including paralysis and darkness of the abdomen as shown now in new supplementary figure (S6 Fig). The graphs show mortality typically continued to increase after 26 hours (ln 137). We have indicated in the text, as showed in the video with our *in vitro* fed flies, that flies surviving longer than 24 hours were unable to fly after feeding on NTBC treated mice.

- *Line 155-159: The text (and figure legends) for figure 2 describe features that are not readily apparent on the included micrographs. Please highlight specific features of interest on figures.*

  Response: We have re-annotated Fig 2 to be more reader-friendly by inserting headings within the image and indicating how the transversal confocal images are linked to the

confocal overview. We have also replaced panel D with a dorsal view of the tsetse eyes, which better shows the differences in eye colour.

- *Line 160-173: as above*

  Response: We have re-annotated Fig 2 as explained above.

- *Line 200-208: It is not clear whether these data were generated with mesotrione or nitisinone. The section heading states NTBC, but supp. Fig. 7 states mesotrione*.

  Response: We apologize for this mistake and thank you for catching it. The data were generated with mesotrione and we have corrected this in the section heading.

- *Line 209-215: state concentration of NTBC, state concentration range of BSA, concentration of sugar (fructose in this case).*

  Response: All concentrations tested are now stated in the methods section (page 25-26, lines: 605-613)

- *Line 217-225: State method of application, vehicle, volume, concentration range applied. See comment above on topical application.*

  Response: This information is now included in the method section (page 27, lines 644-651)

- *Line 218: Provide exact numbers of flies on the graph. Specify which sex was tested, and which line corresponds to which concentration.*

  Response: We have added this information to the legend in Fig.1.

- *Line 240: it's not clear why the authors used p450s from mosquitoes rather than from tsetse. This limits the relevance of these findings.*

  Response: We used an *in vitro* expressed and purified P450 from mosquitoes (CYP6P3) (Müller et al., 2008) as a positive control as this enzyme is involved in resistance to pyrethroids. The microsome extraction protocol is routinely used on mosquitoes so we included mosquito microsomes also as an extraction control. Moreover, tsetse P450s are poorly annotated and not available. Mosquito P450s were included as proxy of representative insect metabolic enzymes as they have a very broad cross-resistance profile.

- *Line 243: define "moderately"*

  Response: *In vitro* studies using human liver microsomes and cDNA-expressed P450 enzymes have shown limited CYP3A4-mediated metabolism (Orfadin Capsules: Summary ofProduct Characteristics. Available at: http://www.ema.europa.eu/docs/en_GB/document_library/ EPAR_-_Product_Information/human/000555/WC500049195.pdf.). However, Neat et. al

(Jason N. Neat, Andrea Wolff, Faraz Kazmi, Pilar Prentiss, David Buckley, Elizabeth M. Wilson, John Bial, Cedo M. Bagi, 2010) investigated the metabolism of NTBC in human liver microsomes and the potential for NTBC to inhibit or induce human cytochrome P450 enzymes. NTBC (at 1, 10 and 100 µM concentrations) was incubated with multiple concentrations of NADPH-fortified pooled liver microsomes (0.5, 1 and 2 mg/mL) for multiple incubation times (0, 30, 60, 120 and 240 min). Despite the wide range of concentrations used, little-to-no loss of NTBC was observed, which suggests that NTBC undergoes insignificant oxidative metabolism by human liver CYP enzymes.

- *Line 267: sugar solutions of NTBC didn't kill tsetse flies either. The authors should test topical application in bees, as possible mortality effects in bees may be unrelated to protein uptake.*

  Response: Testing topical application in bees should be straightforward, but unfortunately the lab of co-author Seth Berribeau (our bee expert) is currently closed due to the COVID-19 situation, so we are unable to run this experiment.

- *…Moreover, the conclusions that NTBC selectively kills blood-sucking insects provided in the discussion (line 386) is not justified given the limited number of insect species tested here and in previous work.*

  Response: We respectfully disagree. In our experiments here with *B. terrestis* (buff-tailed bumblebee: Order Hymenoptera), the bees were continuously provided with NTBC-supplemented sugar for 10 days. Pollen, the natural protein source for bees, was also provided so the bees could feed, *ad libitum* on both as they normally would do in the field. Tsetse flies were continually fed on NTBC-supplemented sugar.

  As this reviewer has highlighted, in previous manuscripts, we tested the effect of HPPD inhibitors on other non-blood feeding organisms such as is *T. castaneum* (red flour beetle: Order Coleoptera) and *O. fasciatus* (large milkweed bug: Order Hemiptera). Neither *T. castaneum* or *O. fasciatus* injected with HPPD inhibitors died, even when they were injected with a dose as high as 20 µg of mesotrione, which is a dose 50 times higher than the dose necessary to kill *R. prolixus* (Sterkel et al., 2016). Tsetse flies are more closely related to sugar-feeding fruit flies (*D. melanogaster:* Order Diptera). *D. melanogaster* HPPD mutants do not show increased mortality (Belfiori-Carrasco et al., 2017). Interestingly, in *C. elegans* the knockdown of *TAT* (Ferguson et al., 2013) and *HPPD* (Lee et al., 2003) enhanced survival. Moreover, HPPD inhibitors have been used as herbicides for more than 20 years (Beaudegnies et al., 2009) and there are no reports related to their toxicity to insects. Altogether, data support that the inhibition of tyrosine catabolism selectively kills blood-feeding arthropods.

- *Line 347: what is the approximate tyrosine content in whole blood (bovine, human). The comparison is interesting.*

  Response: We propose most of the accumulating tyrosine is derived from the degradation of blood proteins.

  To provide an estimate in support for this conclusion:

1. Tyrosine concentrations in human plasma is about 0.06 mM (Borg et al, BMC Gastroenterology 2005, 5:11 doi:10.1186/1471-230X-5-11).
2. Neither haemoglobin, nor albumin (the two more abundant proteins in blood) have a particularly high tyrosine content. Tyrosine (subunits alpha, beta, gamma and epsilon) comprises ~2% of rabbit haemoglobin and 3.46 % of bovine albumin. On average, eukaryotic proteins contain 3% tyrosine (Doolittle, R. F. 1986). Haemoglobin and albumin present a high degree of protein sequence homology between different species, such as humans, birds, cattle and horse. The tyrosine content in albumin of these species varies between 2.97% (horse: 18 Tyr/607 aa) and 3.46% (bovine: 21 Tyr/607aa), while its content in haemoglobin (alpha subunit) varies between 1.41 (horse: 2 Tyr/142aa) and 2.82% (bird: 4 Tyr/142aa).
3. Estimating a 14.5 g% haemoglobin and 4.1 g% albumin concentration in blood, these proteins would generate 6.2 mg Tyr/ml, which translates as ~34 mM of free tyrosine (assuming complete hydrolysis of these proteins). These rough calculations imply that the protein tyrosine pool is approximately over 500 X the plasma free amino acid concentration.

   In summary, these estimates (together with our BSA experiments (Fig. 4)) indicate that the pool of tyrosine involved in the NTBC killing likely comes from protein digestion.

- *Caution is to be encouraged in discussing the unknown environmental safety of NTBC which, given its origin as an herbicide (albeit an agriculturally unsuitable one) may have some environmental impact. See also comment above about beneficial insects.*

  Response: Nitisinone and other members of triketone family were initially developed for agricultural purposes. However, nitisinone failed to reach the final steps of the herbicidal design and development process because its potency (affinity with plant HPPD) was lower than other inhibitors. However, its toxicity profiles were evaluated in many mammals and it was considered to be a safe drug. According to our current and previous results, it selectively kills arthropods when they feed on blood. A single bloodmeal typically exceeds the insect's body weight and dry weight composition of the blood is ~ 85%). This feeding trait and large protein ingestion sets blood feeders apart from nectar feeding insects. It is possible that other organisms feeding on a protein-rich diet, such as the larvae of meat-eating insects such as flesh fly (*Sarcophaga* sp.), would also be affected by the inhibition of HPPD. This hypothesis remains to be tested.

  We agree with the reviewer that using NTBC may have unpredictable environmental impacts, but, as discussed in this manuscript, we present evidence that this strategy using HPPD inhibitors is more environmentally friendly than using the neurotoxic insecticides currently in use. These insecticides are definitely not selective and their extensive use has created insecticide resistant populations that jeopardise agricultural development and public health interventions.

- *The suggestion that nitisinone could be used in a human MDA approach is presented without scrutiny. Given the potential ethical issues of administering a human therapeutic*

en masse with no direct benefit to the individual, I would like to see some discussion of these issues.

Response: As suggested by the reviewer, we included a paragraph in the discussion related to these issues (page 13-14, lines 320-335) that specifically reiterates the reviewer's concerns and suggests how further research in humans must be approached with great caution and only considered under specific circumstances like a disease outbreak scenario. We have also added the following sentence to the discussion to emphasize our position – "Nevertheless, before mass treatment of humans with NTBC is considered, caution should be exercised; it should only be used in regions where trypanosomiasis transmission continues despite best efforts in case management" (page 13, lines 317-319).

- *Topical application of compounds dissolved in a volatile vehicle (e.g. acetone), should not be considered a direct proxy for compound contact on a flag-trap, surface-treated animal, or similar substrate. Acetone disrupts the waxes of the insect exocuticle and, as a result, facilitates traversal of dissolved compounds into the insect hemocoel. While the fact that external exposure to nitisinone is effective is an important finding, some discussion of the limitations of this technique should be included.*

  Response: Agree, although we are not suggesting using acetone for the topical application of NTBC. As a starting point for topical assays, most application protocols with insecticides are performed using acetone as the vehicle. These protocols are used specifically to determine the LD_{50} and other insecticide parameters. To avoid the use of powerful organic solvents like acetone, we aim to investigate NTBC toxicity on tsetse using tarsal assays as our research in collaborations with LITE using mosquitoes has shown that NTBC can be absorbed through insect tarsi (unpublished data). We, however, agree with the reviewer that if used in a field situation, other vehicles for delivery of NTBC should be used should topical application be appropriate. We recommend that NTBC would be best used as an endectocide as topical application requires much higher doses to kill tsetse (our data).

Reviewer #4:

- *The authors use a simple model to estimate the impact of distributing NTBC as an endectocide for HAT, to humans and/or livestock, and predict the dosing interval for NTBC needed to drive R below 1. It's great to see modeling deployed to estimate the potential impact of a new tool like this! However, I have a few concerns with model structure and additional concerns about parameterization:*

  In the model, infection in animals is the same as infection in humans. The model says animal infections are just as transmissible and it is not at all clear that this is the case for T. b. gambiense, which accounts for the vast majority of HAT infections.

  Response: We agree that equivalent tsetse transmission from animals and humans is not appropriate for *T. b. gambiense*. We took the reviewer's recommendation to follow the examples from Rock, Stone, et al. and have only factored transmission between tsetse flies and humans. Following the widely accepted critical role of animals in *T. b. rhodesiense* transmission (Welburn et al 2006 DOI: 10.1016/j.pt.2006.01.011), we

allow for transmission to both animals and humans. Now we present NTBC control impact for both transmission settings.

- *Why exp(-\lambda t^2) instead of exp(-\lambda t) for the drug decay?*

  Response: We have corrected the drug decay function following the reviewer's recommendation.

- *Equation for m_2 suggests effective coverage of 100% for humans (or animals) treated with NTBC. This is unrealistically high. Operationally realistic excellent coverage with mass drug administration, which this is, would probably not exceed even 80%.*

  Response: We have now corrected the coverage rate following the reviewer's recommendation.

- *No evidence that model can capture observed HAT dynamics: no description of model calibration to HAT incidence data or other model validation. Would be great to see this in a revision so that we can be confident that the model is capturing relevant transmission.*

  Response: We definitely agree that fitting models to data is an important step in mathematical epidemiology. But, respectfully, this is the one reviewer comment that we do not think applies to this current manuscript. There are many stages in the development of novel disease control options and, arguably, modelling can help at every stage. At the operational implementation stage, unequivocally, model development needs to be married closely with the control setting. This involves a model of greater complexity that can incorporate local information (extant disease management strategies, seasonality, population age-distribution, bite-rate heterogeneities, etc.) and that can be used to sensibly fit to disease data. Fitting simple models that omit locally important patterns and processes to disease data risks spurious results and interpretations. The model we have selected to determine feasibility of HAT control with the novel endectocide is analogous to the Ross-Macdonald model which has 'played a central role in development of research on mosquito-borne pathogen transmission and the development of strategies for mosquito-borne disease prevention' (Smith et al. 2012 PLoS Pathogens). This simplistic and transparent modelling is more appropriate for the current, early, proof-of-principle stage. Later in its development, models such as those cited by the reviewer, will become more appropriate tools for assessing NTBC deployment strategy. We have added this explanation to the manuscript.

- *Current control methods also rely heavily on treatment, which in recent years has become much less onerous. It's probably fine to ignore treatment in a paper that is very focused on a novel vector control method but it should be acknowledged that no one would pursue mass NTBC distribution unless good case management were already in place.*

  Response: We completely agree with this statement and thank the reviewer for highlighting this. We have thus added the following text to the manuscript: 'It should

be noted that, once its safety is assured, mass treatment of humans with NTBC would likely only be considered an option in regions which have failed to curb transmission despite best case-management efforts". (page 13, lines 317-319)

- *The authors seem unfamiliar with the existing HAT modeling literature from a number of groups, including Kat Rock and Nakul Chitnis, for example:*

  Kat S Rock, Martial L Ndeffo-Mbah, Soledad Castaño, Cody Palmer, Abhishek Pandey, Katherine E Atkins, Joseph M Ndung'u, T Déirdre Hollingsworth, Alison Galvani, Caitlin Bever, Nakul Chitnis, Matt J Keeling, Assessing Strategies Against Gambiense Sleeping Sickness Through Mathematical Modeling, Clinical Infectious Diseases, Volume 66, Issue suppl_4, 15 June 2018, Pages S286-S292,

  Stone CM, Chitnis N. Implications of heterogeneous biting exposure and animal hosts on Trypanosomiasis brucei gambiense transmission and control. PLoS Comput Biol. 2015 Oct 1;11(10):e1004514.

  Response: Sincere apologies for the omission of these important HAT modelling papers – both are now included in the updated version.

Additional references for reviewers' responses

Barrera, R., Carapeto, M., Habela, M., Zaragoza, C., 2010. Electrophoretic pattern of serum proteins in horses with babesiosis. Arch Med Vet 42, 173–178.

Beaudegnies, R., Edmunds, A.J.F., Fraser, T.E.M., Hall, R.G., Hawkes, T.R., Mitchell, G., Schaetzer, J., Wendeborn, S., Wibley, J., 2009. Herbicidal 4-hydroxyphenylpyruvate dioxygenase inhibitors-A review of the triketone chemistry story from a Syngenta perspective. Bioorganic Med. Chem. 17, 4134–4152. https://doi.org/10.1016/j.bmc.2009.03.015

Belfiori-Carrasco, L.F., Marcora, M.S., Bocai, N.I., Ceriani, M.F., Morelli, L., Castaño, E.M., 2017. A Novel Genetic Screen Identifies Modifiers of Age-Dependent Amyloid β Toxicity in the Drosophila Brain. Front. Aging Neurosci. 9. https://doi.org/10.3389/fnagi.2017.00061

Ferguson, A.A., Roy, S., Kormanik, K.N., Kim, Y., Dumas, K.J., Ritov, V.B., Matern, D., Hu, P.J., Fisher, A.L., 2013. TATN-1 Mutations Reveal a Novel Role for Tyrosine as a Metabolic Signal That Influences Developmental Decisions and Longevity in Caenorhabditis elegans. PLoS Genet. 9, e1004020. https://doi.org/10.1371/journal.pgen.1004020

Hall, M.G., Wilks, M.F., Provan, W.M., Eksborg, S., Lumholtz, B., 2001. Pharmacokinetics and pharmacodynamics of NTBC (2-(2-nitro-4-fluoromethylbenzoyl)-1,3-cyclohexanedione) and mesotrione, inhibitors of 4-hydroxyphenyl pyruvate dioxygenase (HPPD) following a single dose to healthy male volunteers. J Clin Pharmacol 52, 169–177.

Jason N. Neat, Andrea Wolff, Faraz Kazmi, Pilar Prentiss, David Buckley, Elizabeth M. Wilson, John Bial, Cedo M. Bagi, M.G. and A.P., 2010. In vitro inhibition and

induction of human liver cytochrome P450 enzymes by NTBC and its metabolism in human liver microsomes. Drug Metab. Rev. 42.

Lee, S.S., Kennedy, S., Tolonen, A.C., Ruvkun, G., 2003. DAF-16 target genes that control C-elegans life-span and metabolism. Science (80-.). 300, 644–647. https://doi.org/10.1126/science.1083614

Lock, E.A., Gaskin, P., Ellis, M.K., McLean Provan, W., Robinson, M., Smith, L.L., 2000. Tissue distribution of 2-(2-nitro-4-trifluoromethylbenzoyl)-cyclohexane-1,3-dione (NTBC) and its effect on enzymes involved in tyrosine catabolism in the mouse. Toxicology 144, 179–187. https://doi.org/S0300483X9900205X

Müller, P., Warr, E., Stevenson, B.J., Pignatelli, P.M., Morgan, J.C., Steven, A., Yawson, A.E., Mitchell, S.N., Ranson, H., Hemingway, J., Paine, M.J.I., Donnelly, M.J., 2008. Field-caught permethrin-resistant *Anopheles gambiae* overexpress CYP6P3, a P450 that metabolises pyrethroids. PLoS Genet. 4, e1000286. https://doi.org/10.1371/journal.pgen.1000286

Orfadin®Capsules: Summary ofProduct Characteristics., n.d.

Sterkel, M., Perdomo, H.D., Guizzo, M.G., Barletta, A.B.F., Nunes, R.D., Dias, F.A., Sorgine, M.H.F., Oliveira, P.L., 2016. Tyrosine Detoxification Is an Essential Trait in the Life History of Blood-Feeding Arthropods. Curr. Biol. 26, 2188–2193. https://doi.org/10.1016/j.cub.2016.06.025
