# Supplementary material for: Repurposing the orphan drug nitisinone to control the transmission of African trypanosomiasis
Source: PLoS Biol. 2021 Jan 26;19(1):e3000796. doi: 10.1371/journal.pbio.3000796 (PMC7837477; doi:10.1371/journal.pbio.3000796)
Supplement: Attachment — Submitted filename: Responses to editorial team.pdf [file pbio.3000796.s024.pdf]

LSTM
LIVERPOOL SCHOOL
OF TROPICAL MEDICINE

Pembroke Place,
Liverpool, L3 5QA, UK
Tel: +44(0)151 705 3100
Fax: +44(0)151 705 3370

www.lstmed.ac.uk

21 November 2020

re: PLoS Formatting Requests for PBIOLOGY-D-20-01398R1

Dear PLoS Biology Team,

Thank you for your guidance during the final stages of manuscript submission. I believe we have incorporated all the changes required with the exception being the author contributions. As you will notice, the "Author contributions" remain at the end of the manuscript as I could not find where I should upload this information on the online submission form.

We have uploaded two high resolution images that represent the blood-feeding lifestyle of the tsetse fly and also a third striking image that more poetically represents the lethality an endectocide could have on tsetse populations, and ultimately disease control.

With regards to creating a press release, our institution's Communications Department will be keen to engage with you on how best to proceed. Therefore, we are unsure at this point as to whether it is best to initially pre-publish the paper online or to wait until the issue is released.

Please let me know if anything outstanding requires my attention.

Sincerely,

Álvaro Acosta Serrano, PhD

Department of Vector Biology
Liverpool School of Tropical Medicine
Tel: +44-151-705-3304

Researching and educating to save lives
A Company Limited by Guarantee. Registered Number 83405, England and Wales. Registered Charity Number 222655.

Athena SWAN
Bronze Award
