# Supplementary material for: Repurposing the orphan drug nitisinone to control the transmission of African trypanosomiasis
Source: PLoS Biol. 2021 Jan 26;19(1):e3000796. doi: 10.1371/journal.pbio.3000796 (PMC7837477; doi:10.1371/journal.pbio.3000796)
Supplement: Attachment — Submitted filename: Responses to the editor.docx [file pbio.3000796.s023.docx]

**Responses to the editor’s and Reviewer #4’s comments and requests**

**Editor:** The control BSA group figure that you provided in the response to reviewers should be included in the manuscript, as readers may have the same concern**.**

**Response:** As suggested, this figure has been included in the manuscript as supplementary Figure S9. Flies were daily fed with either 0.1% fructose-PBS or PBS alone supplemented with 34 mg/ml of BSA). All the figures numbers in SI were changed accordingly. Additionally, the following has been added on page 9 (lines 209-211): “Only a small percentage (~6%) of flies died 24 h after feeding with either PBS-BSA (34 mg/ml) or fructose-BSA without NTBC (S9 Fig.), indicating that the 24 h mortality was due to the addition of NTBC”.

**Editor:** The manuscript should reference the Oliveira lab preprint (bioRxiv [doi.org/10.1101/669747](http://doi.org/10.1101/669747)) on the use of HPPD inhibitors for mosquito control that you allude to in response to reviewer 1 and discuss the implications of those results (it increases the relevance of the work to say if the strategy could be used for mosquitoes control, given their importance as vectors of disease).

**Response:** These references and a brief discussion were added in the Discussion section on page 13 (lines 312-317): “In agreement with our results on tsetse, a recent preprint article from the Oliveira’s group has shown evidence that NTBC is more potent than mesotrione and isoxaflutole against *Aedes aegypti* mosquitoes [33]. Moreover, NTBC was also found to be lethal against *Anopheles* and *Culex*, as well as *Ae. aegypti* strains resistant to neurotoxic insecticides [33], indicating that cross-resistance between NTBC and neurotoxic insecticides is unlikely”.

**Editor:** Your metabolomics dataset should be deposited in an appropriate public repository, such as MetaboLights, and the accession number included in the Data Availability statement (in the online article submission form) and were relevant in the manuscript.

**Response:** Done. The following has been added on page 29-30 (lines 712 -714): “Metabolomics data (6 replicates) was deposited into the MetaboLights repository (https://www.ebi.ac.uk/metabolights/) under accession number MTBLS2166”.

**Editor:** Figure 2A needs a scale bar

**Response.** Done. The following has been added to the legend of Fig 2A (line 895)**: “**(A) Fat Body. Scale bars = 100 mm (control) and 80 mm (treated)”. We have also added scale bars to all other panels in Fig 2 where appropriate.

**Editor:** Units need to be provided for scale bar in the legend to SI 1

**Response:** The units are provided in Fig S1 legend to read “Scale bars (branch lengths) correspond to the mean number of amino acid substitutions per site on the respective branch”.

**Editor:** Statistical information (n, whether the mean or median is plotted, what kind of error bars, etc) is missing in the legend of figure 1, for the box-and-whisker plot data in 3B, and for Suppl Figure 4

**Response:** Done. The legend of Suppl. Fig. 4 was corrected. In all figures, the data are shown as mean ± SEM. The legend for figure 1D was corrected since, by mistake, it was indicated the data was shown as median ± SEM.

All the survival curves presented in the manuscript are shown as Kaplan-Meier survival curves, as it is indicated in the “Statistical analysis” section (page 34, lines 831-838). Because there are many survival curves in the manuscript, we highlight this in the method section.

Regarding Fig 3B, we have represented statistical significance as the following symbols: ns = P > 0.05, * = P ≤ 0.05, ** = P ≤ 0.01, *** = P ≤ 0.001, **** = P ≤ 0.0001, which has been added to the figure legend (page 37, lines 910-911).

We are also including a supplementary file (S1 Data; excel spreadsheet) indicating the number of flies used in each replicate of all the experiments presented in the figures. We included in the method section the phrase “Numerical source data underlying all figures can be found in Supplementary Data 1.” (page 35, lines: 839-840).

**Editor:** As there are no length restrictions, please include all of the Material and Methods information in the main text. The detailed explanation of the mathematical model can be left in SI, and the file labelled accordingly, but the other two subheadings should be moved to the main text.

**Response:** The manuscript has been modified according editor’s suggestions. Only the mathematical model was left in SI.

**Editor:** You may be aware of the PLOS Data Policy, which requires that all data be made available without restriction: <http://journals.plos.org/plosbiology/s/data-availability>. For more information, please also see this editorial: <http://dx.doi.org/10.1371/journal.pbio.1001797>

Note that all individual quantitative observations that underlie the data summarized in the main and supplementary figures of your paper need to be made available as source data. Each figure legend should include a statement indicating where the numerical source data for the figures can be found (e.g. Numerical source data underlying this figure can be found in XXX). You can provide source data in one of the following forms:

1) Supplementary files (e.g., excel). Please ensure that all data files are uploaded as 'Supporting Information' and are invariably referred to (in the manuscript, figure legends, and the Description field when uploading your files) using the following format verbatim: S1 Data, S2 Data, etc. Multiple panels of a single or even several figures can be included as multiple sheets in one excel file that is saved using exactly the following convention: S1_Data.xlsx (using an underscore).

2) Deposition in a publicly available repository such a GitHub. If you choose this option, please also provide the accession code or a reviewer link so that we may view your data before publication.

Regardless of the method selected, please ensure that you provide the individual numerical values that underlie the summary data displayed in all of the main and supplementary figures that present graphs, as they are essential for readers to assess your analysis and to reproduce it:

NOTE: the numerical data provided should include all replicates AND the way in which the plotted mean and errors were derived (it should not present only the mean/average values).

Please also ensure that figure legends in your manuscript include information on where the underlying data can be found, and ensure your supplemental data file/s has a legend that makes it easy to understand.

**Editor:** The Supplementary Figures need to be provided as individual files

**Response:** We have referenced all source data as appropriate and have embedded raw data in a comprehensive excel spreadsheet	entitled S1_Data, deposited metabolomics data in the online repository called *MetaboLights* (accession number MTBLS2166). The manuscript text has been amended to include these additions. Figure legends have been checked for clarity and all Supplementary Figures exist as individual files.

Reviewer expertise:
**Reviewer #2**: tsetse physiology and trypanosomiasis control
**Reviewer #3**: strategies to prevent malaria transmission; mosquito vector control; malaria pathogenesis
**Reviewer #4**: mathematical modelling of malaria transmission and effects of antimalarial strategies

Comments to authors:
**Reviewer #2**: Thank you to the authors for their comprehensive response to my and the other reviewers critiques. The manuscript is much improved and the issues raised in my previous review have been addressed.

**Response:** We are pleased the reviewer can appreciate the improved manuscript and thank them for their insight, which lead to a more thorough work. **Reviewer #3**: This is a greatly improved resubmission. The authors have done a good job at addressing all comments, adding more information when needed and more comprehensively explaining their findings, as well as their significance and limitations. I just have some minor revisions to add:

**Response:** Thank you for your comment. We have implemented your suggested revisions.

Line 320: provide references for this statement

**Response:** Done. We added the following reference for this statement (Ref 22. Page 13, line 331). 22. Foy BD, Kobylinski KC, Silva IM da, Rasgon JL, Sylla M. Endectocides for malaria control. Trends Parasitol. 2011;27: 423–428. doi:10.1016/j.pt.2011.05.007

Line 391: I think the word 'albeit' doesn't fit here

**Response:** We agree. It has been removed.

Lines 408-430. I particularly appreciated the more detailed discussion of possible emergence of tsetse resistance. However, insects are capable of evolving multiple resistance mechanisms besides target site and metabolic resistance, and I encourage the authors to acknowledge that eventually flies would develop resistance to their compound if it was used as monotherapy, as they suggest in line 438. 
**Response:** We agree with the reviewer that insects are capable of evolving multiple resistance mechanisms. In this manuscript we only	suggest, based on our findings and data previously published, that two important mechanisms commonly conferring resistance to insecticides (P450s-mediated detoxification and target-site mutations) are unlikely to generate resistance toward HPPD inhibitors in insects. We included a new phrase on page 18, lines 442-443 according to the reviewer’s suggestion: “*However, since insects are capable of evolving multiple resistance mechanisms, they could eventually develop resistance to NTBC if used as monotherapy*”.

**Reviewer #4**: Two main points on the modeling section:
1. Clarity on model assumptions
2. Relevance of NTBC to public health

I get what the authors are saying re: model validation. Their goal is to use the simple Ross-Macdonald and see what happens if mortality of the vector is increased through endectocide. But the authors need to do a much better job being explicit about the assumptions of their model because the modeling section of the paper is key to showing that that NTBC has relevance for public health.

**Response:** We have added further details around these methods and included a new figure (Fig S14) to improve the model’s description and to highlight important assumptions. Please see our responses to the specific points below.

**Reviewer**: It's somewhat unclear to me how the Re values with intervention are being calculated. Is m2(t) plugged into the analytical solution to R to derive an Re(t)? Then are the Re values shown in Fig 5 a mean Re? Or is Re(t) being estimated directly from the simulation with a method like EpiEstim? I'm a little bit confused by the supplemental methods description.

**Response:** The simulations are conducted over a 1-day time step. Meaning, from the day of dosing where maximal additional vector mortality occurs following bites, the killing effect wanes a little each day. In this way, m2 decreases slightly each day and this reduces its impact on Re each day (calculated using the analytical expression for Re). Over time, Re returns to pre-intervention levels (i.e. R0). We’ve added additional explanation in the main text (page 11-12, lines 280-282. Page: 34, lines 821-823) and supplementary materials to clarify this point (“Therefore, the *Re* as plotted in Fig 5 (for *T. b. rhodesiense*) and in Fig S15 (for *T. b. gambiense*) is the *Re* averaged over the treatment cycle. To show how these summary plots relate to temporal dynamics, we have plotted example dynamics for when *T. b. rhodesiense* is controlled using monthly dosing of both humans and livestock with NTBC. The many lines shown in Fig S14 are the simulated results for a random assortment of bloodmeals (split between humans, livestock and wildlife”).

**Reviewer**: Then are the Re values shown in Fig 5 a mean Re? Or is Re(t) being estimated directly from the simulation with a method like EpiEstim? I'm a little bit confused by the supplemental methods description.

**Response:** We apologize. The reviewer’s first assumption is right – it is a mean Re over the period of time between doses. This is now described more fully in the Supplementary Information document: “*As the killing efficacy decreases between endectocidal applications, Re returns to its pre-control level (i.e., R0). Therefore, the Re as plotted in Fig 5 (for T. b. rhodesiense) and in S15 Fig (for T. b. gambiense) is the Re averaged over the treatment cycle*.”

To further improve clarity of the modelling results we also generated another figure showing the temporal dynamics (S14 Fig) and the following description in the SI section of how the temporal dynamics relate to the figure displayed in the main text: “*To show how these summary plots relate to temporal dynamics, we have plotted example dynamics for when T. b. rhodesiense is controlled using monthly dosing of both humans and livestock with NTBC*.”

**Reviewer**: Either way, does this mean that the modeling assumes that NTBC distribution continues indefinitely? I think this is a key assumption that should be explicitly mentioned to help readers interpret the modeling results. Furthermore, there's a key assumption of R0 = 1.1 that is not stated explicitly in the main text.

**Response:** The temporal dynamics of control achieved using NTBC are	newly added to the SI section (S14 Fig). We have also added a more detailed explanatory text in SI methods on how the impact of NTBC on Re displayed in the main article is its averaged impact over the period of time between doses. We are a little confused by the question of whether NTBC would be distributed indefinitely. Without achieving elimination, vector and/or disease control methods would typically need to be sustained for as long as their benefits were desired. If this question pertains to the feasibility of sustaining NTBC treatment indefinitely at a specific site, then we are in agreement with the reviewer and have included an acknowledgement in the main text (page 12. lines 287-292) that this type of strategy is better suited for short pulses (e.g. as part of an integrated strategy for achieving elimination in a given setting).

**Reviewer**: My read of Fig 5 and Fig S13 is that NTBC in its current form is not an operationally relevant potential vector control: monthly MDAs (or even 3-monthly) at 80% coverage aren't going to be sustainable for long enough to interrupt transmission, and the modest dip of Re to just below 1 in most of the searched parameter space would indicate that a long program of MDAs would be needed. Given the low case numbers of both rhodesiense and gambiense HAT, WHO is focusing on improved diagnostics and treatments to interrupt transmission (gambiense) or eliminate as a public health problem (rhodesiense). The modeling results for NTBC don't suggest that it would drive a shift in HAT policy toward vector control.

**Response:** In locations where humans and livestock comprise the large majority of tsetse fly blood sources (likely where HAT persists currently), our simulations show that an approximate halving of R0 can be achieved. This achievement is more apparent now with the inclusion of a new figure of the control dynamics in the supplementary materials (apologies as previously it was obscured by averaging the control achieved over the	time between treatments). This result notwithstanding, should NTBC in its current formulation be used, we would be in agreement with the reviewer’s read of the situation – it would still not constitute a viable standalone HAT elimination strategy. However, in the main text we state that NTBC should **only** ever comprise a component of an integrated strategy for eliminating HAT. And, the importance of using integrated approaches, including novel vector controls, for achieving elimination was one of the general conclusions of the third WHO meeting of stakeholders on the elimination of gambiense HAT in Geneva between the 18th and 20th of April 2018, which was attended by representatives from national sleeping sickness control programmes, groups developing new tools to fight HAT, international and nongovernmental organisations involved in HAT control, industrial partners and funding agencies (10.1371/journal.pntd.0006925). The conclusions of our study therefore seem to align well with current the WHO strategy. We include this additional explanation in our main text (page 18, lines 448-451) and thank you for highlighting	this concern.
